# Applying YOLOv6 as an ensemble federated learning framework to classify breast cancer pathology images

**DOI:** 10.1038/s41598-024-80187-7

**Published:** 2025-01-30

**Authors:** Chhaya Gupta, Nasib Singh Gill, Preeti Gulia, Noha Alduaiji, J. Shreyas, Piyush Kumar Shukla

**Affiliations:** 1 Department of Computer Science and Applications, Maharshi Dayanand University, Rohtak, India; 2 Department of Computer Science, College of Computer and Information Sciences, Majmaah University, 11952 Al Majmaah, Saudi Arabia; 3 Department of Information Technology, Manipal Institute of Technology Bengaluru, Manipal Academy of Higher Education, Manipal, Karnataka 576104 India; 4 Department of Computer Science and Engineering, University Institute of Technology, Rajiv Gandhi Proudyogiki Vishwavidyalaya (State Technological University of Madhya Pradesh), Madhya Pradesh, Bhopal 462033 India

**Keywords:** Federated learning (FedL), YOLOv6, Breast cancer, Transfer learning, ResNet-50, Inception-V3, Cancer, Computational biology and bioinformatics, Ecology, Health care, Medical research

## Abstract

The most common carcinoma-related cause of death among women is breast cancer. Early detection is crucial, and the manual screening method may lead to a delayed diagnosis, which would delay treatment and put lives at risk. Mammography imaging is advised for routine screening to diagnose breast cancer at an early stage. To improve generalizability, this study examines the implementation of Federated Learning (FedL) to detect breast cancer. Its performance is compared to a centralized training technique that diagnoses breast cancer. Although FedL has been famous as a safeguarding privacy algorithm, its similarities to ensemble learning methods, such as federated averaging (FEDAvrg), still need to be thoroughly investigated. This study examines explicitly how a YOLOv6 model trained with FedL performs across several clients. A new homomorphic encryption and decryption algorithm is also proposed to retain data privacy. A novel pruned YOLOv6 model with FedL is introduced in this study to differentiate benign and malignant tissues. The model is trained on the breast cancer pathological dataset BreakHis and BUSI. The proposed model achieved a validation accuracy of 98% on BreakHis dataset and 97% on BUSI dataset. The results are compared with the VGG-19, ResNet-50, and InceptionV3 algorithms, showing that the proposed model achieved better results. The tests reveal that federated learning is feasible, as FedAvrg trains models of outstanding quality with only a few communication rounds, as shown by the results on a range of model topologies such as ResNet50, VGG-19, InceptionV3, and the proposed Ensembled FedL YOLOv6.

## Introduction

Artificial Intelligence has become an essential topic in the field of healthcare. It is critical to receive the results promptly because a delayed diagnosis can lead to delayed treatment, worsening the patient’s condition before the cancer spreads and treatment becomes insufficient. Doctors can improve patients’ chances of survival with the appropriate treatment. An AI clinician may provide a second opinion to a doctor uncertain about a cancer diagnosis. It helps doctors act more quickly and save lives. These AI doctors can be installed on embedded devices and linked to phones or IoT devices to expedite the diagnosis. To facilitate collaboration between various medical agencies without disclosing sensitive patient data, federated learning is utilized where data privacy is subject to stringent restrictions^1^. Without gaining access to each other’s private patient information, federated learning (FedL) enables several users to pool their local AI models (which they can share) and integrate them into a global model.

FedL, which holds the data samples locally rather than transferring them, intends to train a machine-learning algorithm across numerous decentralized nodes. To prepare a decentralized model in a FedL configuration, three primary obstacles must be overcome that are: (i) system and analytical heterogeneity, (ii) data security, and (iii) optimization of models globally^2^. In this framework of FedL, these three problems are addressed for breast cancer categorization. Heterogeneity in systems and data is the first challenge. Different systems create images using intensity profiles that are noticeably different for the same imaging modality. To address this issue, a single dataset has been shared between all the clients, reducing heterogeneity among other models. The cryptographic and transfer learning techniques address the second obstacle to data privacy^3^. The cryptographic process helps purposefully perturb the local model’s parameters before being uploaded to the server for integration. It helps to secure the data by tuning its parameters, while the transfer learning technique helps save time and improves the overall performance of the proposed model. To handle the third obstacle, where the central server is responsible for integrating all the local models with all their local updates, a novel ensembled FedL YOLOv6 is proposed.

This study aims to explore the application of FedL to object identification problems and examine the similarities between federated learning methods, such as Federated Averaging (FEDAvrg), and ensemble learning techniques. In this study, researchers employ FedL’s communal nature to improve the model’s overall recognition capabilities, creating a more generalized and stable federated learning YOLOv6 model. This study compares the performance of the well-known YOLOv6 algorithm for breast cancer detection to the conventional centralized training method taught through ensemble FedL with numerous clients. To achieve this, researchers suggest an approach in which federated training is performed by several clients, each of which has access to the identical dataset used for centralized training. Random sampling without replacement is used to distribute the data among clients. Using the YOLOv6 model, the performance of FedL for object detection in an ensemble scenario is evaluated.

Even though the tasks have been performed with centralized systems till now, there are some differences in why federated learning should be used instead of centralized systems. Federated Learning and centralized training techniques differ significantly in the context of pathological image classification, particularly in terms of data privacy, scalability, and data distribution. In centralized training, all data is aggregated and stored in a central location, such as a server or cloud infrastructure, for model training. This centralized approach poses privacy risks, requiring the sharing of sensitive medical data from multiple sources with a central entity. Meanwhile, Federated Learning, on the other hand, allows model training to be performed locally on individual devices or nodes without sharing raw data. Only model updates or gradients are exchanged between the central server and participating nodes. This decentralized approach preserves data privacy by keeping sensitive data localized and encrypted during training. Centralized training can face scalability challenges, especially with large-scale datasets or many contributing institutions. Processing and storing massive amounts of data centrally can strain computational resources and lead to communication bottlenecks. Federated Learning offers inherent scalability advantages by distributing the training workload across multiple nodes or devices. Each node independently trains a local model using its data, reducing the computational burden on the central server. This distributed approach enables federated learning to scale efficiently with the number of participating institutions or devices. Federated Learning offers significant advantages over centralized training techniques in pathological image classification, particularly regarding data privacy, scalability, data distribution, and regulatory compliance. By preserving data privacy, leveraging distributed data sources, and facilitating collaborative model training, federated learning enables the development of more robust and privacy-preserving pathological image classifiers. In a traditional centralized training approach, data from various hospitals and institutions would need to be aggregated into a central server. This involves sharing sensitive medical images (e.g., mammograms, histopathological slides) across institutions, which raises significant privacy concerns. With FedL, individual institutions can train local models on their own patient data, and only model updates (gradients or weights) are shared with a central server. This way, no raw data ever leaves the institution, preserving patient confidentiality and complying with data protection regulations. When combined with privacy-preserving techniques such as homomorphic encryption, FedL can ensure that even the model updates are encrypted, providing an additional layer of security. In medical settings, data is often siloed across different hospitals, institutions, and research centers. Centralized training requires the aggregation of all data in one place, which is often impractical due to privacy and regulatory constraints. With FedL, each institution can contribute to model training while keeping the data locally. This enables the use of data from various geographically distributed locations without the need for large-scale data transfers, which can be slow and costly. FedL allows distribution of training across multiple institutions, each with its own computational resources. This decentralization reduces the burden on a single central server and allows parallel computation, which can speed up the overall training process.

The main contributions of this paper are:

- Designing an ensembled FedL-based YOLOv6 for breast cancer recognition using a central server that works as a weight integrator and helps integrate various local models. FedAvrg (Federated Averaging) technique is used to upgrade weights. Different hospitals and clinics can widely use this approach.
- A new 64-bit homomorphic encryption and decryption algorithm is proposed based on the diffusion of Shannon’s theory. The algorithm also uses operations like XOR, XNOR, and swapping. The secret keys are generated using these operations. A new encryption and decryption algorithm is proposed because most users do not use encryption and decryption techniques. Existing encryption techniques make use of traditional generators to generate secret keys. The existing approaches use a single key for all data.
- Using Transfer Learning and finetuning the parameters of YOLOv6 improves the performance and efficiency of the overall proposed model. Various models like ResNet-50, VGG-19, and Inception v3 were used to compare the proposed model’s performance. The results clearly show that the proposed ensembled FedL-based Yolov6 model, when trained on the BreakHis dataset, achieved 6% better results than centralized models. When trained on the BUSI dataset, it achieved 4% better results than centralized models. The results showed that the accuracy achieved was approximately 98%.

The remaining portions of the paper are arranged as follows: The literature review is covered in “Literature review”, the methodology is covered in “Methodology”, the experimental outcomes and discussions are presented in “Experimental results and discussions”, and the study is concluded in “Conclusion”.

## Literature review

### Application of deep learning to breast cancer diagnosis

Globally, breast cancer is the primary cause of cancer-related mortality among women. Numerous computer-aided technologies have been suggested for determining the presence of breast cancer. Typically, mammographic analysis of breast tumor material is the first step in diagnosis. Several classifiers are subsequently used for particular nucleus features. The nuclear image’s structure offers a repeatable pattern for the histological cytopathological diagnosis of malignancy.

In today’s breast cancer research, deep-learning algorithms are mainly used to detect and diagnose breast tumors^4^. Lou Luyang et al.^5^ extensively reviewed deep-learning-based breast cancer classification techniques. They also discussed the challenges and potential avenues for future work. Saber Abeer et al.^6^ profounded a novel transfer learning-based deep-learning model for diagnosing breast cancer. The proposed model was compared with VGG-19, ResNet50, Inception V3, and Inception V2 models. The model achieved good results but is not suitable for real-time applications. Huan-Jung Chiu et al.^7^ profounded a processing method for classifying breast cancer with nine attributes. They also implemented Principal Component Analysis (PCA) for dimension reduction. The proposed method achieved an accuracy of 86.97%. Accuracy can be increased further by using transfer learning techniques.

Rishav Singh et al.^8^ presented a transfer learning-based VGG-19 model for diagnosing imbalanced breast cancer at an early stage. ImageNet dataset that consists of 277,524 images was used to train the model. The model achieved good results but can be further improved by pruning. Kiran Jabeen et al.^9^ proposed a framework using a haze-reduced contrast removal technique to enhance the images. The researchers used the EfficientNet model as a base model. The experiment was carried out on CBIS-DDSM and INbreast datasets, and the model achieved 95% accuracy. Samraj Dhivya et al.^10^ suggested a genetic algorithm-based histogram equalization approach for improving the visual aspects of medical images. The process is a data mining technique and achieved an accuracy of 94% while diagnosing breast cancer.

Jiang Shu et al.^11^ profounded a supervised functional principal component analysis (sfPCA) method for feature extraction. The authors also proposed an eigenvalue decomposition technique. The process was applied to the dataset from Joanne Knight Breast Health Cohort at Siteman Cancer Center, and the suggested method helped the model achieve better accuracy. Fei Yan et al.^12^ presented an ensemble classifier and feature weighting algorithm for classifying breast cancer in mammography images. The model is designed using Bagging, KNN, and eigenvalue classification models. The method was trained on DDSM and MIAS datasets.

Sivamurugan et al.^13^ profounded a novel hybrid model that uses an optimized deep-learning architecture to diagnose breast cancer. The model uses Three pre-processing techniques: morphological operation, histogram equalization, and median filtering. The model achieved about 96% accuracy. Hanife et al.^14^ reveal what optimal combinations of pre-processing techniques are available with the classification technique for diagnosing breast cancer at an early phase. The classification methods, such as random forest and SVM, achieved the best results.

Abdulrahman Abbas et al.^15^ suggested a novel Dual Transfer Learning approach in the field of medical imaging. The approach is applied with VGG16, Xception, ResNet50, and MobileNetV2 models. The Xception model achieved the highest accuracy of 96.83% on the ISIC2020 dataset and 99% accuracy on the ICIAR2018 dataset. Executing fine-tuning on the models by teaching the last layers of images can be used to improve the performance of the tasks of classifying breast cancer images due to the similarity of the images in the histological structure, which can be used to extract features that are like the features of breast cancer.

Tariq Mahmood et al.^16^ developed deep-learning algorithms to improve breast lesion detection, localization, risk assessment, and classification, aiming to reduce false positives on human intervention and tackle slow convergence rates. The Chaotic Leader Selective Filler Swarm Optimization (cLSFSO) method is a significant development that effectively detects breast-dense lesions by extracting textural and statistical features. The model is based on VGGNet and ResNet-152. The model achieved good results. Tariq Mahmood et al.^17^ proposes a novel deep learning-based convolutional neural network (ConvNet) for classifying breast malignant tissues. The model achieved an accuracy of 98%. Tariq Mahmood et al.^18^ improves the accuracy of the previously proposed model ConVNet to 97.8%.

### Ensemble learning with federated learning (FedL)

The merging of large amounts of data can help machine learning models. In the medical domain, access to data is severely constrained due to concerns about user privacy and data confidentiality. Therefore, privacy-preserving decentralized interactive machine-learning approaches are appropriate when developing intelligent medical diagnosis systems. Several frameworks exist, including federated transfer learning, horizontal FedL, and vertical FedL, to solve this problem. Recently, FEDL was brought to light due to the requirement for service providers in numerous industries, such as innovative healthcare and smart cities, to communicate sensitive data.

Tran Khoa et al.^19^ presented a FedX framework for monitoring health in cloud-based networks. This framework uses a random sampling technique to balance the data. This framework uses an Encoded Depth Convolutional Network (EDCN) for classification. The study lacks robustness, as histopathological datasets across institutions vary significantly due to patient demographics, imaging techniques, and labeling criteria. Duy-Dong et al.^20^ profounded a multi-modal FedL for predicting the Air Quality Index (AQI). They merged machine learning models with a federated learning approach. The model fails to handle the increase in computational cost.

Holger Roth et al.^21^ suggested a robust federated learning framework based on deep learning for classifying breast cancer with mammographic images. Amelia Jimenes et al.^22^ profounded a novel memory-aware curriculum learning for diagnosing breast cancer. The researchers used curriculum learning in the federated learning setting. Zezhong et al.^23^ suggested a novel federated learning model merged with CNN to predict breast cancer. The model achieved an accuracy of 90% but lacks generalizability. Bless Agbley et al.^24^ profounded a recurrent neural network with federated learning. The researchers used Gabor kernels for feature extraction, and the model achieved 80% accuracy. The accuracy of the model can be further improved in the future by merging transfer learning and pruning.

Integrating ensemble learning with federated learning can benefit both approaches, allowing effective learning from distributed datasets while reaping the rewards of numerous learners’ predictive abilities. Jeny Hamer et al.^25^ introduced a boosting algorithm called FedBoost to reduce costs. FedBoost is an ensembled algorithm whose main objective is to minimize communication costs on both sides, server-to-client and client-to-server. Sen Lin et al.^26^ suggested a platform-based integrated learning framework to achieve real-time edge intelligence. Researchers examined the federated meta-learning algorithm’s convergence on node similarity at the target edge under relaxed conditions.

Terrail Du et al.^27^ profounded an ensembled federated learning model for predicting triple-negative breast cancer. The model is trained with annotations based on time. Tan et al.^28^ introduced a federated learning algorithm merged with CNN and SMOTE techniques. SMOTE helps minimize the oversampling problem. Zengqian et al.^29^ suggested a variation-aware federated learning framework for reducing the images of all clients to image space with the help of a generative adversarial network. Savita Kumbhare et al.^30^ profounded a hybrid federated learning model with meta-heuristic deep learning models for predicting breast cancer. The model was trained on the DDSM dataset and achieved 95% accuracy. Shi Jun et al.^31^ profounded a pseudo-data-based supervised federated learning framework for improving diagnostic accuracy in pathological images.

From the above study, it is concluded that several limitations commonly arise in research papers when implementing federated learning with a pruned YOLO model on histopathological breast cancer images. These limitations affect model performance, generalizability, and practical deployment, which researchers often encounter. Histopathological datasets across institutions vary significantly due to patient demographics, imaging techniques, and labeling criteria. This non-identically distributed (non-IID) data affects model performance because federated learning often assumes balanced IID data. Although federated learning enhances data privacy, model updates can still leak information about local datasets, especially with high-dimensional data like histopathological images. Many studies lack real-world validation, relying on simulated data splits instead of real hospital data distributions. Some of these challenges are worked on in this study.

In this study, YOLOv6 is considered the detection algorithm for breast cancer images. YOLOv6 is an advanced version of Yolov5, and it is easy to make it learn migration learning. The methodology used in a prior study is aligned with the choice of YOLOv6 as the object detection system^32^. While others have developed more complex concepts, none have examined the fundamental similarities between federated and ensemble learning.

### Motivation

The technological motivation for combining Federated Learning (FedL) with YOLOv6 for histopathological breast cancer images stems from several key needs in the healthcare and AI fields. Breast cancer diagnosis through histopathological image analysis requires accurate, efficient, and secure deep learning models, which can be effectively addressed through federated learning and the advanced object detection capabilities of YOLOv6. Breast cancer histopathology datasets are often stored in hospitals, research centers, and clinics. Sharing sensitive patient data across institutions for centralized training poses significant privacy risks and can violate data protection regulations. Federated learning enables training on decentralized data without transferring sensitive patient data. The model is trained locally on the histopathology images stored at each institution, and only model updates (gradients) are shared with a central server. This preserves privacy while allowing collaborative training across multiple institutions. YOLOv6 is a high-performance, real-time object detection model that can be integrated into the FedL framework to identify and localize cancerous regions in histopathological images. The FedL approach ensures that YOLOv6 is trained on diverse datasets from multiple institutions without exposing sensitive data.

Histopathological images of breast cancer are complex and vary significantly between patients, hospitals, and regions. It’s difficult for a single institution to have enough data to train a highly generalized and robust model, especially for rare cancer subtypes or stages. Federated learning allows multiple hospitals or research centers to collaborate and pool their datasets without centralizing the data. This leads to a more diverse and representative dataset, enabling the development of a robust model that generalizes well across different populations, image acquisition techniques, and cancer subtypes. YOLOv6 can benefit from the diversity in the datasets distributed across various institutions. Federated learning allows it to learn from diverse histopathological image patterns (such as varying stain types, cancer stages, and tumor morphology), leading to better cancer detection accuracy and generalization.

Histopathological images are often large, and traditional classification models like DenseNet or EfficientNet can be computationally expensive and slow in inference, especially in resource-constrained clinical environments. YOLOv6 is optimized for real-time object detection, offering both speed and accuracy. This is crucial in medical applications where quick and accurate diagnosis can lead to timely treatment decisions. YOLOv6’s efficiency makes it well-suited for detecting tumors or suspicious regions in large histopathological images at scale. YOLOv6’s model updates are shared between clients and the central server in federated learning. Its lightweight architecture ensures that updates (in terms of parameters or gradients) are minor, reducing the communication overhead. This makes it feasible to train YOLOv6 in a federated setting, even in environments with limited bandwidth or computer resources, such as smaller hospitals.

In real-world scenarios, data from different institutions can be heterogeneous, i.e., non-IID (non-independent and identically distributed). For example, histopathological images may come from different scanners, be stained differently, or represent various patient demographics and cancer subtypes. Federated learning inherently addresses data heterogeneity by training models locally on each institution’s data, allowing the model to capture institution-specific characteristics. When model updates are aggregated, the central model benefits from the combined knowledge across all institutions, thus improving generalization across different types of data. YOLOv6’s real-time object detection and localization capabilities allow it to adapt well to non-IID data. With federated learning, YOLOv6 can learn from a wide variety of image distributions while being deployed in different hospitals with different histopathological data. This improves its robustness to variations in image acquisition and staining protocols. A pseudocode for federated learning with YOLOv6 for histopathological breast cancer images is depicted as:
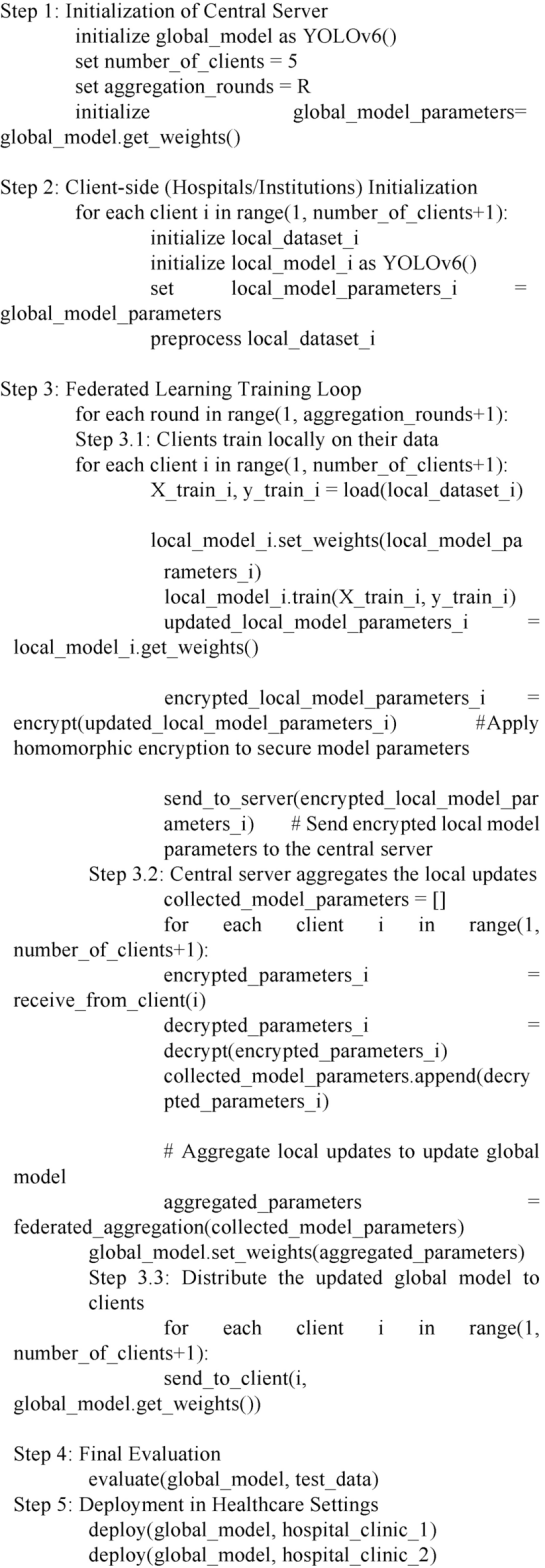


## Methodology

This section describes the suggested federated ensemble learning technique in detail by categorizing clients as weak learners. The primary dataset is divided into various client-specific subgroups. This division helps Federated Averaging (FedAvrg) to empower fragile clients to develop traits and characteristics by utilizing global models. This approach helps create a robust global model that exhibits superior generalization abilities and helps classify target items. InFedLuenced by the ensemble aspect of FedL, a practical object detection model has been created to classify breast cancer. The proposed model comprises a Yolov6 algorithm trained on BreakHis and BUSI datasets identical to the dataset utilized by centralized Yolov6 for training. This approach divides the datasets into equal partitions based on the number of clients compared to traditional FedL. Each client trains the federated global model, where it is imperative to guarantee active involvement in all communication rounds. This methodology helps create an innovative combination of FedL and ensemble learning.

Federated learning is a machine learning technique in which each device does local model training independently while the training data is dispersed among several devices or nodes. The updates to the model are then aggregated to produce a global model. This setup allows for collaborative learning without centralizing sensitive data. The number of nodes involved in federated learning can vary depending on the application and infrastructure. Typically, federated learning setups can involve thousands to millions of devices, such as smartphones, IoT devices, or servers, each contributing to the training process. However, the number of nodes can be tailored based on factors like the scale of the dataset, computational resources, and the desired level of collaboration. This study uses 5 clients; hence, there are five nodes. One of the most essential aspects of federated learning is data privacy. Several techniques are employed to ensure data privacy during the learning process. This study uses data encryption and decryption techniques for data privacy.

- Local Model Training: In federated learning, each device uses local data for model training. Privacy is maintained because the raw data is never removed from the device.
- Differential Privacy: Before aggregating the local updates, techniques like differential privacy can introduce noise or perturbations to them. This helps in preventing the extraction of sensitive information from individual data samples.
- Encryption: Data can be encrypted before transmission or during computation to prevent unauthorized access. Homomorphic encryption ensures privacy by enabling computations on encrypted material without decrypting it.
- Secure Aggregation: Techniques like cryptographic protocols or secure multi-party computation (MPC) can safely aggregate model updates. This ensures that the updates are combined without revealing individual contributions.
- On-Device Processing: Instead of sending raw data to a central server, computations can be performed locally on devices, and only aggregated updates or model parameters are transmitted. This reduces the risk of exposing sensitive information during data transmission.
- Data Filtering and Anonymization: Before participating in federated learning, data can be filtered or anonymized to remove personally identifiable information (PII) or sensitive attributes. This reduces the risk of privacy breaches during model training.

BreakHis dataset is divided into training, validation, and testing sets^33^. 7909 breast tumor tissue images obtained from 82 clients using various magnification factors comprise the Breast Cancer Histopathological Image (BreakHis) dataset. It has 700 × 460 pixel samples, 2480 benign and 5429 malignant, with 3-channel RGB and 8-bit resolution in each channel. Ten percent is set aside for testing, ten percent is for validation, and eighty percent is for training. Breast Ultrasound Images (BUSI) dataset consists of 1312 ultrasound scans of breast cancers^34^. It has 500 × 500 pixel samples, 891 benign samples, and 421 malignant samples. Ten percent is set aside for testing, ten percent is for validation, and eighty percent is for training. The images used to train the centralized model are distributed to different clients, while the dataset is distributed to other clients. To make the approach more understandable, only five clients are considered for this experiment, and they are provided with identical test and validation sets used in the centralized model.

Preprocessing the BreakHis dataset to train YOLOv6 involves several key steps to convert the raw histopathological breast cancer images into a format suitable for object detection. YOLOv6 is an object detection model, and it requires annotated images with bounding boxes around the objects of interest (in this case, tumor regions). However, BreakHis is primarily a classification dataset, so the preprocessing step involves creating bounding box annotations. YOLOv6 is an object detection model, and it requires annotated images with bounding boxes around the objects of interest (in this case, tumor regions). However, BreakHis is primarily a classification dataset, so the preprocessing step involves creating bounding box annotations. If bounding box annotations are not available, they need to be created manually using annotation tools like LabelImg tool. The annotator would draw bounding boxes around the tumor regions and assign the appropriate labels (e.g., benign, malignant). YOLOv6 expects input images of a specific size. The images need to be resized and normalized to match the input dimensions of the YOLOv6 model. All images are resized to a fixed dimension (e.g., 640 × 640 pixels) to ensure uniformity across the dataset. This is a crucial step since YOLOv6 operates on a fixed input size for efficient detection. Pixel values are often scaled to a range of [0, 1] or standardized to have a mean of 0 and standard deviation of 1. This helps in faster convergence during training.

To enhance model generalization and handle the data imbalanc**e** in BreakHis (as malignant cases are usually more prevalent than benign), data augmentation techniques are applied. In this study, data augmentation techniques have been applied to local clients. The clients perform data augmentation techniques on their local data before encrypting and sending updates. This helps reduce the imbalance locally without needing central access to the raw data. To tackle this issue, some local data augmentations are made on clients, such as random rotations, Flips, and scaling of images. Clients can augment minority-class data before encryption. This can help improve the balance of local datasets and produce more representative model updates. Though it does not entirely solve the problem of imbalance at the global level, it helps maintain the model’s performance at a decent level. Same steps are taken for BUSI dataset.

**Fig. 1:**
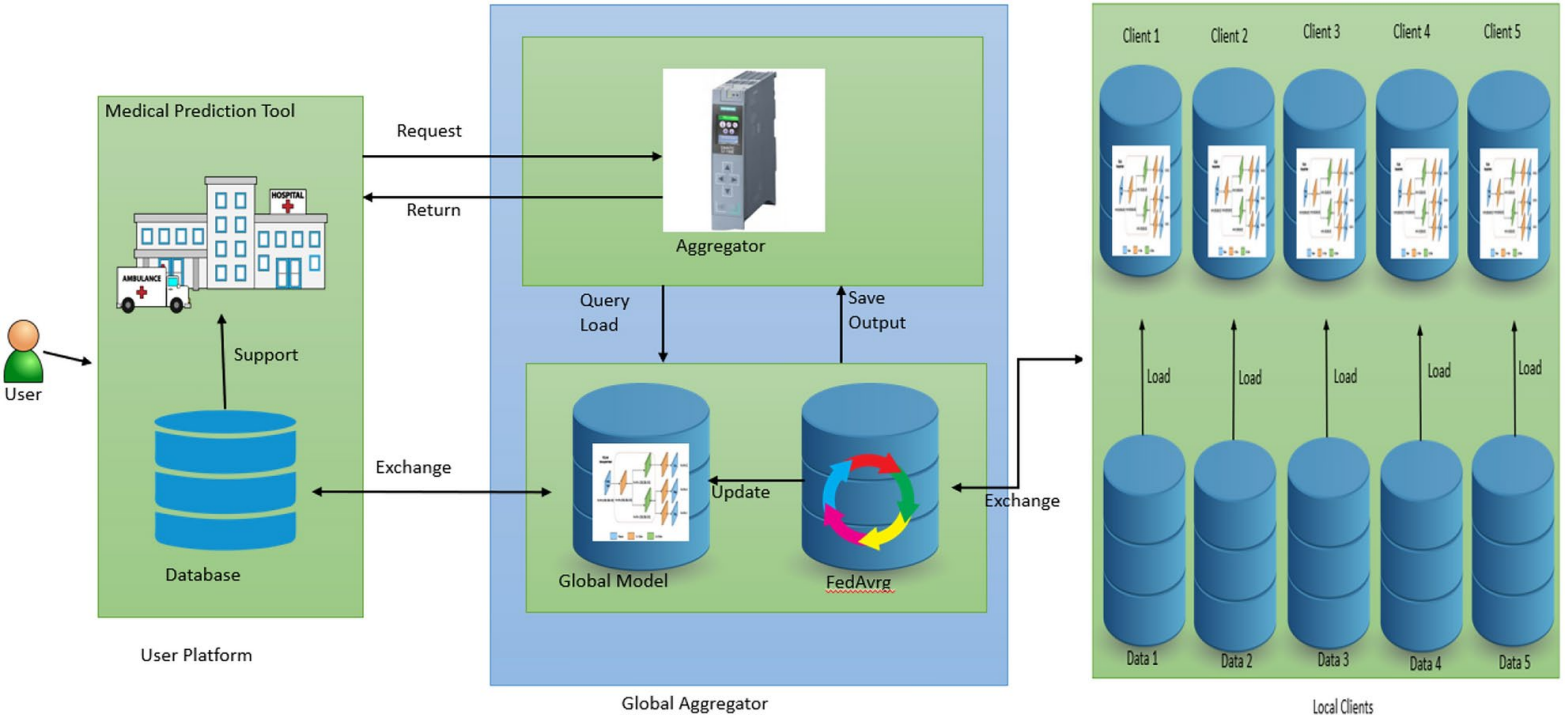
Ensembled FedL architecture for breast cancer image classification showcasing the flow of data from database to aggregator, global model, and local clients.

**Fig. 2:**
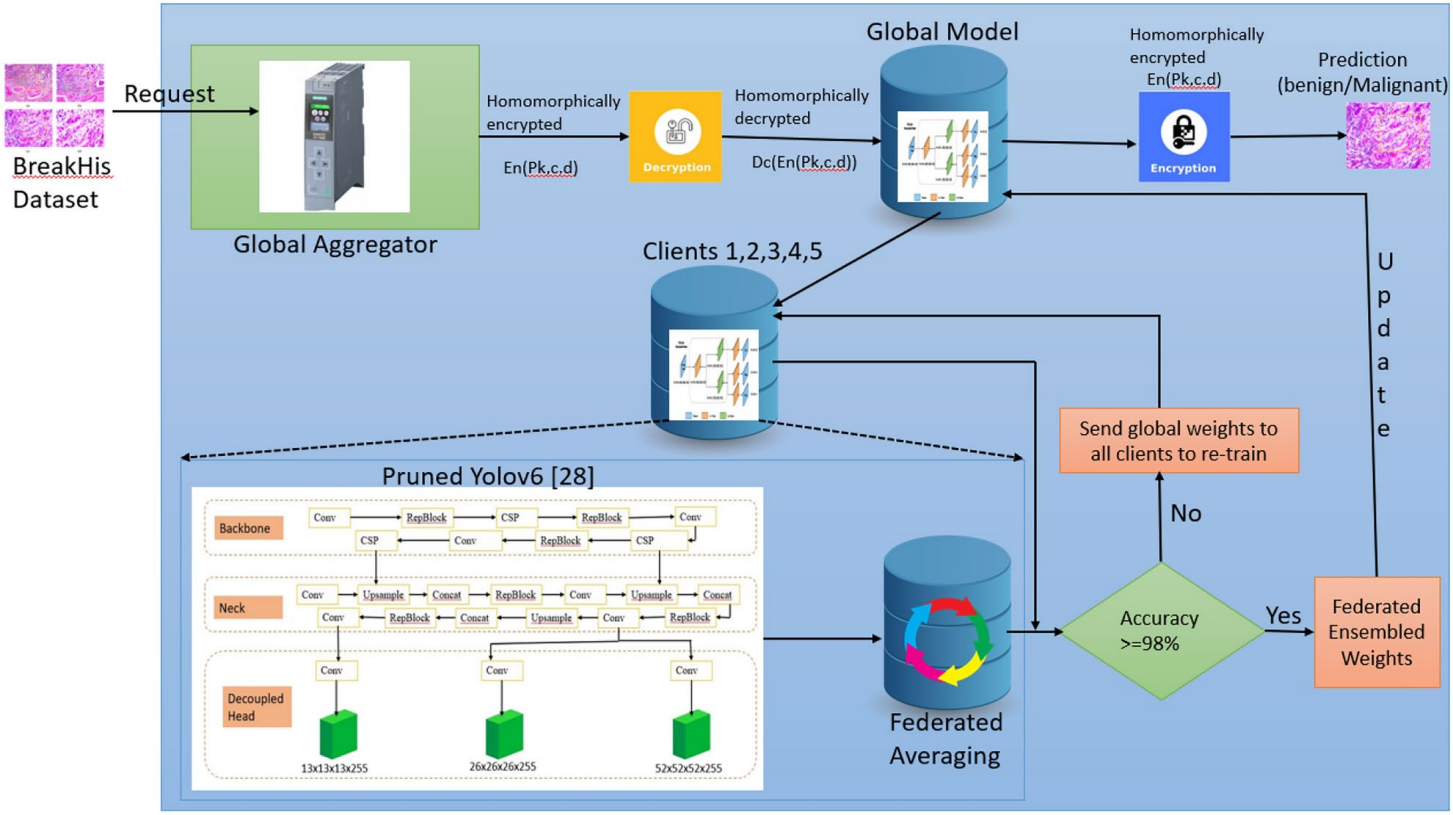
The working of aggregator, global model, and local clients is depicted showcasing how different techniques are combined together to form a single model.

The proposed ensembled FedL model for classifying breast cancer tumors is illustrated in Fig. 1. The model has three parts: first is the global aggregator that aggregates the weights in each communication round and updates the global model, the second is the local clients where hospitals are connected to local models, and the third is user platform that is divided into medical imaging prediction tools and Database. Users can access prediction requests, and the database provides the required data. The global model comprises an aggregator and the global model container. The aggregator aggregates the weights in each communication round and performs the task scheduler function. The aggregator helps schedule the encrypted request for the worldwide model. The aggregator process is depicted in Fig. 2. The global model first decrypts the homomorphically encrypted request^35^, shares it with the desired client’s model, and again returns the homomorphically encrypted prediction results. If the predicted results are unsatisfactory, the global model is optimized with the generated federated ensembled weights during training. The international model comprises a pruned YOLOv6 model^28^ and a federated averaging technique. The FedAvrg technique is explained later in this section.

All the local clients have the pruned YOLOv6 model, and the federated averaging technique installed on them. Each client trains the local model with the local data, and all the encrypted ensembled federated weights are uploaded to the global model. The global model again uses a federated averaging technique to aggregate all the parameters received from the clients. The global model is updated with all the parameters, and the updated model parameters are shared with all the clients. This process repeats itself until the training is complete. The user initiates a homomorphically encrypted request and sends it to the global aggregator module. The aggregator then analyses the request and maps it to the global model. The global model chooses the appropriate client that carries out the prediction and returns the encrypted results and the ensembled federated weights. All these parameters are used to update the global model.

Utilizing an ensemble federated learning YOLOv6 framework for breast cancer detection addresses several challenges inherent to this medical imaging task. Medical data is extremely sensitive and governed by stringent privacy laws, including data from breast cancer imaging. Several healthcare facilities can work together on model training with federated learning without exchanging raw patient data. By encrypting and decentralizing the data across multiple nodes and using techniques like differential privacy, the privacy of patient information is preserved while still enabling collaborative model training. Breast cancer classification datasets often suffer from class imbalance problems, where benign cases may significantly outnumber malignant cases. Additionally, imaging data may vary in quality and presentation across different healthcare institutions. Ensemble learning with federated approaches allows models trained on diverse datasets to be combined, leveraging the strengths of individual models and improving generalization across various patient populations and imaging protocols. Ensemble learning combines predictions from several other models to improve the detection model’s resilience and generalization. YOLOv6, known for its real-time performance and accuracy, serves as the base architecture for each model in the ensemble. Federated learning distributes the computational burden of model training across multiple healthcare institutions or nodes, making it more scalable and resource-efficient than centralized approaches. Each node trains its YOLOv6 model on local data, and the updates are shared and aggregated, reducing the need for large-scale data transfer and centralized computational resources.

The overall architecture uses the following steps:

- The central data is split into 'n' exclusive datasets, where 'n' is the total number of local clients and shufFedLed with YOLO annotations.
- All the local clients use the same YOLOv6 architecture as the global model, use the same federated averaging technique, and have consistent parameters (like number of epochs, batch size, learning rate, and optimizer).
- Each local client is trained using its local dataset for several epochs; the last epoch weights are saved. Each client uses the federated averaging technique to optimize the desired results. This step helps to reduce the pressure of optimization on the global model. Due to this step, the global model has to update itself only once after each communication round.
- The saved weights of each local client are used to update the global model. The global model then performs the federated averaging technique to optimize itself.
- The accuracy of the global model is calculated by testing this model on the testing dataset.
- The steps repeat themselves for better results.

The rest of this section discusses all the concepts in detail.

### Homomorphic encryption and decryption

The foundational components of digital healthcare platforms and systems are trust and privacy. Confidence is anticipated to grow between the stakeholders in the digital healthcare ecosystems, including patients, healthcare professionals, health authorities, and healthcare system providers. In terms of privacy, the following medical data is among the most important and needs to be protected:

- Address, social security number, birth date, and bank account number are only a few examples of the patient’s data.
- Patient’s medications, equipment, processes, and medical and psychiatric services.
- Information about the medical facility, practice, or staff members offering medical and psychological services.

All the details mentioned above are personal and private for the patient and must be protected against cyber threats. Homomorphic encryption and decryption are considered to resolve the threat issue against sensitive data. Data encryption is now standard practice for both businesses and individuals. With the use of the encryption technique known as homomorphic encryption, text that has been encrypted or ciphered can be immediately subjected to mathematical operations without the need for any decoding.

This study uses homomorphic encryption over traditional encryption techniques as it offers a stronger privacy guarantee than traditional encryption methods and is easier to implement than more complex alternatives. Homomorphic encryption enables secure collaboration across multiple healthcare institutions while protecting sensitive patient data, making it an ideal solution for privacy-preserving machine learning in the healthcare domain. Homomorphic encryption is considered a superior option for federated learning in breast cancer imaging for several vital reasons, particularly compared to other encryption methods. The unique properties of homomorphic encryption allow for performing computations on encrypted data without decrypting it, making it highly suitable for privacy-preserving and secure collaborative learning in sensitive healthcare settings like breast cancer diagnosis. The primary concern is maintaining the privacy of patient data, as medical images (such as mammograms) contain sensitive information. Homomorphic encryption allows for computation on encrypted data (e.g., model training and inference) without exposing the raw data to the central server or other participants in federated learning. This ensures patient privacy is preserved, even during the model training process. Traditional encryption methods, such as symmetric or asymmetric encryption (e.g., AES, RSA), do not allow for operations on encrypted data. The data must first be decrypted to perform any kind of computation (e.g., model training in federated learning). This introduces privacy risks, as decrypted data could be exposed to the central server or intermediaries, potentially violating patient confidentiality, especially in highly regulated environments like healthcare. In federated learning, each client (e.g., a hospital or medical institution) trains a model on local data (e.g., breast cancer imaging) and shares the encrypted model updates with the central server. With homomorphic encryption, the server can directly aggregate these encrypted model updates without decrypting them. This means the server can perform operations like secure model averaging or gradient aggregation while keeping the individual client data encrypted throughout the process. Other encryption techniques do not support this ability to perform computations on encrypted data. For example, standard encryption would require the model updates to be decrypted for aggregation, exposing sensitive information.

Homomorphic Encryption is a cryptographic technique that allows computations directly on encrypted data without decrypting it. When decrypted, the result of the computation matches the result if the operations had been performed on the plaintext data. This is especially useful in privacy-preserving applications like federated learning. The fundamental idea behind homomorphic encryption is to allow operations like addition and multiplication to be applied to ciphertexts so that, when decrypted, the result matches the operation applied to the plaintexts. The fundamental property is:

1 $${E}_{{m}_{1}}\otimes {E}_{{m}_{2}}=E({m}_{1}\circ {m}_{2})$$

where, ${E}_{{m}_{1}} and {E}_{{m}_{2}}$ are ciphertexts of plaintexts of ${m}_{1}and {m}_{2}$, $\otimes$ is the operation which could be either ‘ + ’ or ‘*’. $\circ$ represents the corresponding operation in the plaintext domain.

In simple terms, a homomorphic encryption system includes the following components:

- Public and private keys (P_k_, S_k_) are generated. The public key is used for encryption, and the private key is used for decryption.
- A plaintext m is encrypted using the public key P_k_, resulting in a ciphertext c.

To perform addition or multiplication on encrypted data, the operations are performed directly on the ciphertexts. The resulting ciphertext ccc is decrypted using the private key S_k_ to recover the plaintext result.

The computations’ outputs are also encrypted, and when decoded, they yield equal or almost identical results^35^. This is why this study considers homomorphic encryption, as it helps process data without disclosing the original data.

If c and d are actual data, Pk is the encryption key, then,

2 $${\text{f}}\left( {{\text{c}},{\text{d}}} \right) = {\text{Dc}}\left( {{\text{En}}\left( {{\text{Pk}},{\text{c}}} \right),{\text{En}}\left( {{\text{Pk}},{\text{d}}} \right)} \right)$$

where f() is the function applied to existing data.

Homomorphic encryption allows for private third-party computing and storage. This makes data encryption possible while processing in commercial cloud environments. Here, various homomorphic encryption methods are covered:

- When a homomorphic operation, such as addition or multiplication, is only supported partially, it is referred to as partially homomorphic encryption.
- When operations such as addition or multiplication are considered a limited number of times, it is referred to as somewhat homomorphic encryption.
- When multiple gates of unbounded depths are supported in an arbitrary circuit, it is called leveled fully homomorphic encryption.
- When operations such as addition and multiplication are fully supported an infinite number of times, it is called fully homomorphic encryption.

This study uses a new Fully Homomorphic Encryption (FHE), allowing XOR, XNOR, and swapping operations on encrypted data for infinite times. For generating keys, the following steps are performed:

Step 1: Two big primes a and b are chosen in a way, (ab. n = a*b. ω (n) = (a − 1)*(b − 1)), where n = no. of clients and ω is Euler’s coefficient.

Step 2: Integer i is selected such that 1 < i < ω(n), gcd(i, ω(n)) = 1(coprime).

Step 3: k = i-1 mod ω(n).

Step 4: Public key K1 is a combination of (i, n).

Step 5: Private key K2.

Algorithm 1 shows the overall process of homomorphic encryption, and Algorithm 2 shows the homomorphic decryption process.

**Algorithm 1:**
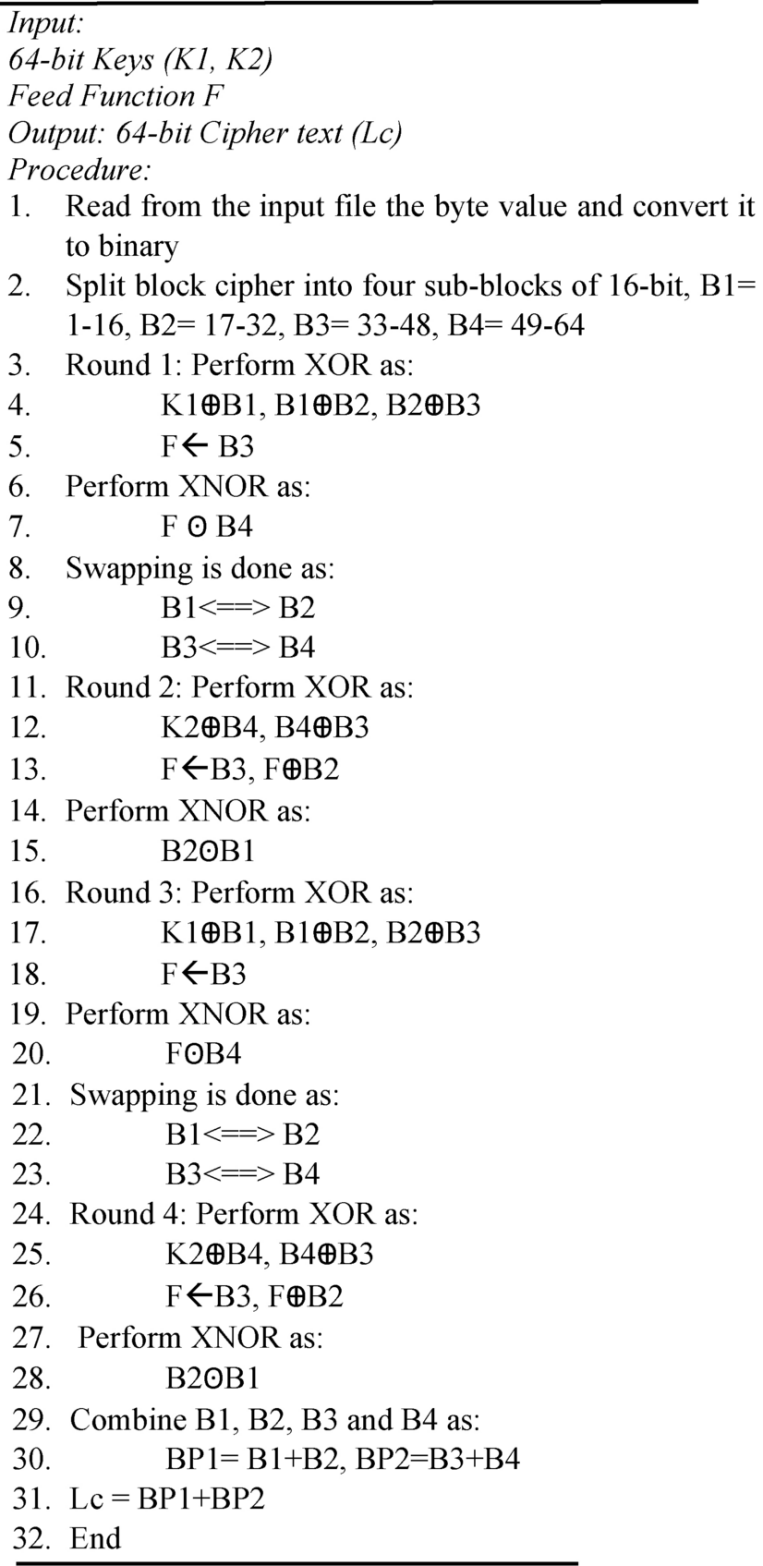
Homomorphic encryption process.

A new encryption and decryption algorithm is proposed because most users do not use encryption and decryption techniques.

Merging Homomorphic Encryption with Federated Learning is an advanced approach to enhance data privacy and security in decentralized machine learning systems. By integrating HE, federated learning systems can ensure that model updates and gradients remain encrypted during transmission and aggregation, protecting user data from potential breaches. In standard Federated Learning, model updates (gradients or parameters) are transmitted from local devices (clients) to a central server, aggregating them to update a global model. While this approach ensures that raw data stays on local devices, it still exposes model updates to the server, which could be analyzed to infer sensitive information. It can enhance privacy by ensuring these model updates remain encrypted. The server can perform computations (aggregation) on encrypted updates without decrypting them, thus maintaining privacy and confidentiality. When integrated into federated learning, homomorphic encryption allows the central server to aggregate client-encrypted model updates without seeing actual updates in plaintext. This can be broken down into the following steps:

Step 1: Local Training and Encryption—Clients train a local model on their private data. Instead of sending plaintext updates (e.g., model gradients or parameters) to the central server, each client encrypts their local model update using a homomorphic encryption scheme (typically public-key encryption). This encrypted update is then transmitted to the server.

Step 2: Encrypted Aggregation on server—The central server receives encrypted updates from multiple clients. Using the homomorphic properties of the encryption scheme, the server aggregates these encrypted updates (e.g., summing gradients or averaging model parameters) without decrypting them. This step preserves the privacy of each client’s update, as the server never has access to the raw updates.

Step 3: Decrypting the aggregated result—The aggregated encrypted update is sent back to the clients once the aggregation is complete. The clients decrypt the aggregated update using their private key to reveal the global model update. The decrypted global update is then applied to the global model, which is repeated for subsequent training rounds.

Mathematically, combining homomorphic encryption and federated learning revolves around performing operations (addition and multiplication) on encrypted data, which are analogous to the ones needed in FedL. In this study, an additive homomorphic encryption scheme has been employed where, each client i encrypts their local model update ΔW_i_ as E(ΔW_i_). The server receives the encrypted updates E(ΔW_1_), E(ΔW_2_), …, E(ΔW_n_) from n clients. Using the homomorphic property of addition, the server computes the global model updates without decrypting individual updates as:

3 $$E\left(\Delta {W}_{global}\right)=E(\Delta {W}_{1}+\Delta {W}_{2}+\dots +\Delta {W}_{n})$$

This aggregated encrypted update $E\left(\Delta {W}_{global}\right)$ is sent back for decryption, and the global model update $\Delta {W}_{global}$​ is applied.

Merging homomorphic encryption with FedL ensures that by keeping model updates encrypted, even during aggregation, clients can be sure that their data and updates remain private, protecting sensitive information. Since the server never sees the raw updates, it cannot infer sensitive information from them, even if compromised, and hence, it boosts the system’s security. This approach can help organizations comply with privacy regulations by ensuring that sensitive data is not exposed.

Despite its benefits, combining Homomorphic Encryption with FedL presents several challenges: Homomorphic encryption is computationally expensive, especially for complex models with significant parameters. Ciphertexts generated by homomorphic encryption schemes are typically much more significant than plaintexts. This increases the communication cost between clients and the server, which is already a bottleneck in federated learning. The proposed technique uses batching to pack multiple model parameters into a single ciphertext to overcome homomorphic encryption’s computational and communication overheads. This significantly reduces the number of encryptions and operations required. By handling multiple parameters together, batching reduces computation and communication costs. The key idea is to use polynomial rings or modular arithmetic to encode multiple plaintext values into a single object, which can then be encrypted and manipulated efficiently. This technique has the following steps:

Step 1: Encoding plaintext polynomially—The core idea behind batching is to represent multiple plaintext values as coefficients of a polynomial, which is then encrypted as a single ciphertext. This is typically done using the Chinese Remainder Theorem (CRT)^36^. For example, to encrypt a vector of n plaintext values [x_0_, x_1_, x_2_, …, x_n-1_], the plaintext vector is encoded into a polynomial as follows:

4 $$P\left(X\right)={x}_{0}+{x}_{1}X+{x}_{2}{X}^{2}+\dots +{x}_{n-1}{X}^{n-1}$$

where the polynomial P(X) represents the entire vector of values, each coefficient of this polynomial corresponds to a plaintext value.

Step 2: Plaintext modulus and Polynomial rings—Homomorphic encryption schemes typically operate over a modular arithmetic system. The plaintext values x_i_ are encoded as elements in a finite field ${\mathbb{Z}}_{q}$ (integers modulus q).

The polynomial P(X) is constructed over a ring of polynomials ${\mathbb{Z}}_{q}\left[X\right]/({X}^{n}+1)$, which means that all operations are modulus of a special polynomial (typically ${X}^{n}+1$). This helps keep the polynomials’ size bounded, allowing efficient arithmetic operations on encrypted data.

Step 3: Encrypting batched polynomials—Once the plaintext values have been encoded into the polynomial P(X), the entire polynomial is encrypted as a single ciphertext. The encryption is done using the public key of the homomorphic encryption scheme.

Step 4: Homomorphic operations on batched ciphertext—Once the data is encrypted in batched form, homomorphic operations (e.g., addition, multiplication) can be performed directly on the ciphertexts. These operations are applied to the encoded polynomials, which means that the operations on the encrypted values correspond to operations on the individual plaintext values. These operations are performed without decrypting the ciphertexts. For example, if there are two ciphertexts C_1_ = E(P_1_(X)) and C_2_ = E(P_2_(X)), the homomorphic addition produces:

5 $${C}_{total}={C}_{1}+{C}_{2}=E({P}_{1}\left(X\right)+{P}_{2}\left(X\right))$$

The proposed homomorphic encryption method cannot cope with the data imbalance problem. It is not designed to manage data imbalance directly because its primary focus is encryption and privacy rather than managing data distribution or characteristics. Homomorphic encryption allows addition, multiplication, and sometimes other operations on encrypted data but does not understand the data distribution. Homomorphic encryption prevents direct access to the underlying data. As a result, any central authority or aggregation server cannot detect or correct data imbalance, and it processes encrypted updates as if all data is equally distributed. When models are trained on imbalanced data under Homomorphic Encryption, they tend to overfit the majority class. Clients with majority-class-dominated data will generate model updates biased toward that class. If many clients have similarly imbalanced data, the global model may become highly biased as it aggregates these skewed updates, amplifying the data imbalance problem. There are potential strategies to deal with data imbalance in traditional machine learning (e.g., re-sampling, re-weighting loss functions, synthetic data generation, SMOTE), but applying these in homomorphic encryption environments is challenging. While homomorphic encryption does not address data imbalance, there are some indirect ways to handle the issue in federated learning. In this study, data augmentation techniques have been applied to local clients. The clients perform data augmentation techniques on their local data before encrypting and sending updates. This helps reduce the imbalance locally without needing central access to the raw data. To tackle this issue, some local data augmentations are made on clients, such as random rotations, Flips, and scaling of images. Clients can augment minority-class data before encryption. This can help improve the balance of local datasets and produce more representative model updates. Though it does not entirely solve the problem of imbalance at the global level, it helps maintain the model’s performance at a decent level.

Existing encryption techniques make use of traditional generators to generate secret keys. The existing approaches use a single key for all data. The proposed Homomorphic encryption process implements Boolean operations such as XOR, XNOR, and swapping to increase the diffusion complexity of encryption. This algorithm uses 64-bit encryption keys K1 and K2 in four encryption rounds. Each round performs mathematical operations of XOR, XNOR, and swapping to produce diffusion complexity. Only four rounds are considered to improve the encryption process’s efficiency. Each round involves 16 bits of data for execution. This algorithm provides mixed operations in various algebraic functions, such as XOR, XNOR, F, and addition operations, to make the attackers’ job more challenging. Encryption is a method for transforming data from a recognizable kind into an understandable one utilizing diffusion techniques. Decryption transfers data from a format users can comprehend with the necessary authority. The algorithm works on four rounds of encryption to reduce energy usage, and each round uses basic Boolean operations to increase data security. The output from each round serves as the next round’s input to produce the cipher text (Lc). The F Feed-Function technique ensures a complex dependency of output bits on input bits by utilizing various non-linear and linear processes. The global model encrypts the results received from the clients and its results and then produces the prediction results.

The decryption process decrypts the cipher text Lc produced during the encryption process. The global model decrypts the encrypted data and then shares it with all the clients. Algorithm 2 provides the overall process for decryption.

**Algorithm 2:**
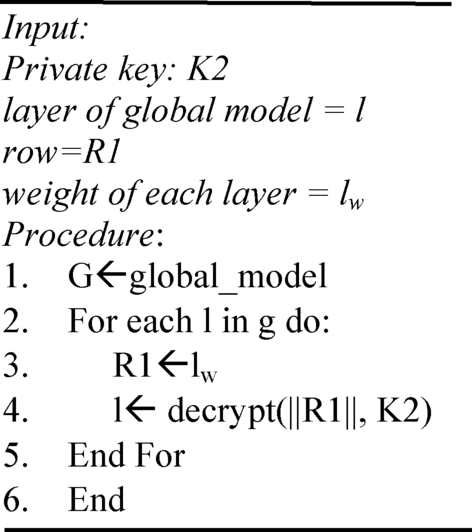
Homomorphic decryption process.

A homomorphic encryption technique makes it possible to do calculations on encrypted material without having first to decrypt it. Because it allows computations to be done on sensitive data while still encrypted, homomorphic encryption is very helpful for maintaining data privacy in federated learning. This lowers the possibility of disclosing sensitive information. Here’s how the proposed homomorphic encryption and decryption algorithm contributes to preserving data privacy in this study:

- Secure Data Transmission: In federated learning, data from individual clients is typically transmitted to a central server or among clients for aggregation and model updates. Encrypting the data using homomorphic encryption before transmission protects sensitive information from potential eavesdroppers or adversaries who may attempt to intercept the data during transmission.
- Privacy-Preserving Aggregation: Homomorphic encryption allows the central server or aggregator to perform computations on the encrypted data without decrypting it first. This enables privacy-preserving aggregation of model updates or gradients from multiple clients. The central server can aggregate the encrypted updates, compute the aggregated result in encrypted form, and then decrypt the final result to obtain the aggregated model update without exposing the individual contributions from each client.
- Data Privacy on Untrusted Servers: In federated learning scenarios where the central server or aggregator is not fully trusted, homomorphic encryption provides additional protection for the clients’ data. Since computations are performed on encrypted data, the server never has access to the plaintext data, reducing the risk of data exposure or unauthorized access.

Overall, the proposed homomorphic encryption and decryption algorithm enhances data privacy in federated learning by allowing computations to be performed on encrypted data without compromising the confidentiality of the underlying information. It enables secure and privacy-preserving collaboration among distributed participants while mitigating the risks of sharing sensitive data in federated learning environments.

### Federated averaging technique

In this study, the Federated averaging (FedAvrg) technique is implemented^37^. Traditionally, the federated learning algorithm generates encryption and decryption keys based on security parameters. Still, in this algorithm, the encrypted public key K1 and decrypted private key K2 are generated using the steps described in “Homomorphic encryption and decryption” and are used in this algorithm.

Federated Averaging (FedAvrg) is one of the most fundamental and widely used optimization algorithms in federated learning (FedL). It serves as the mechanism to aggregate updates from distributed clients (e.g., hospitals or medical institutions) and update a global model without requiring centralized access to the clients’ local data. This technique is crucial when applying federated learning to deep learning models like YOLOv6, especially in sensitive domains like medical imaging, including breast cancer diagnosis. It is the algorithm that enables federated learning to work efficiently across distributed nodes (clients). The key idea is to perform local model training on each client using their private datasets and then send the locally trained model parameters (weights) to a central server for aggregation. The server computes the weighted average of these updates, resulting in a global model that is then shared back with the clients. Each client (e.g., a hospital) trains the model (YOLOv6) on its local dataset. This involves using local data for several iterations to update the model’s parameters (weights) based on the task at hand (e.g., detecting breast cancer regions in histopathological images). Once the local training is completed (usually for a specified number of epochs), each client sends its updated model parameters (e.g., weights) to the central server. The central server aggregates the model parameters from the clients using a weighted average. The weight typically corresponds to the number of data points each client has contributed. After aggregation, the server sends the updated global model parameters back to the clients. Each client then updates its local model with the new global weights and continues with another round of local training.

When implementing federated learning with YOLOv6, the FedAvrg technique can be adapted to handle object detection in a distributed manner across clients. Each client trains a local version of the YOLOv6 model on its dataset. In the context of medical imaging, this could involve breast cancer detection using histopathological or mammographic images. Each client runs its dataset through the YOLOv6 model, making predictions for object detection (e.g., identifying tumor regions in breast cancer images). The loss function in YOLOv6 combines object classification, localization, and bounding box regression errors. Based on the loss, the model weights are updated using optimizers like Adam. After training locally for a set number of epochs or mini-batches, the client sends the updated model parameters (weights) to the central server. The server receives the weights from all participating clients. Each client may contribute a different number of data points, so a weighted average is computed to aggregate the updates. The server computes the global model parameters by averaging the client updates, weighted by the number of samples each client used during local training. After aggregation, the server updates the global YOLOv6 model with the new parameters. The global model is then sent back to each client for the next training round.

The clients train for G number of epochs with pruned YOLOv6 model several times and update the model’s parameters Pw. These updated parameters are sent back to the global model for updation. After the final epoch, the ensembled federated weights of the last epoch are also shared with the global model. The global aggregator aggregates all the updated parameters and the ensembled federated weights to update the global model and to obtain the aggregated parameters Paw. These aggregated weights are shared with each client for their updation. The steps are repeated till the federated training is complete. Algorithm 3 provides the overall procedure for the federated averaging process.

**Algorithm 3:**
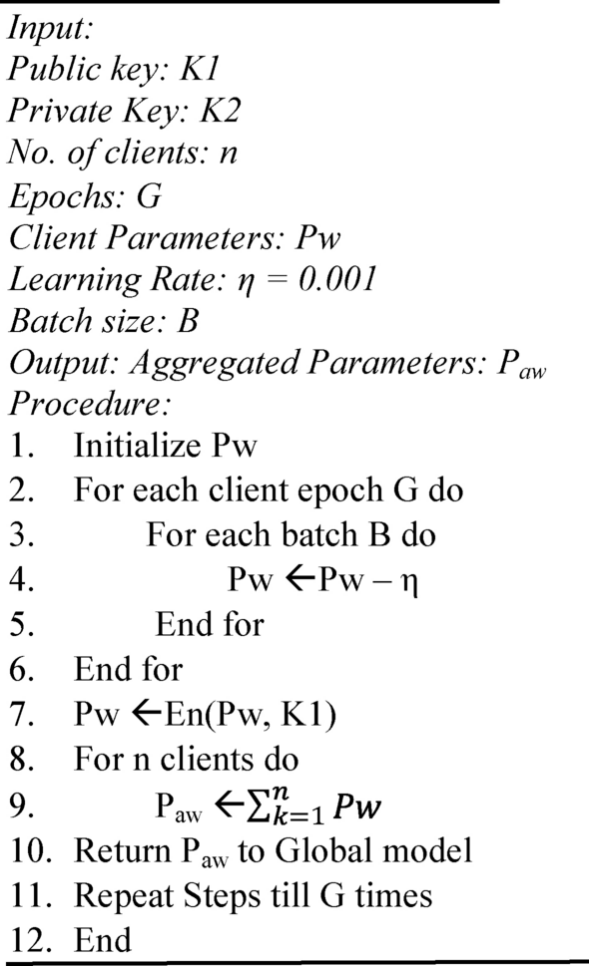
FedAvrg process.

### Local client training

This section describes how each local client is trained using the pruned YOLOv6 model and federated averaging technique. Algorithm 4 shows the overall process of initializing and training the local client. The local classifiers, Ci, are trained with their respective Database, dbi. The weight matrix of model |Mw| is encrypted and is again shared with the local aggregator for aggregation.

**Algorithm 4:**
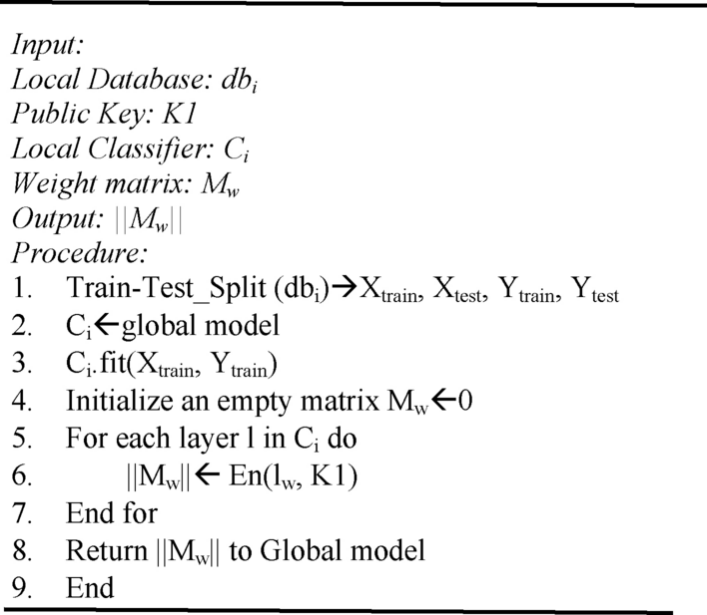
Local client initialization and training.

### Model aggregation

**Fig. 3:**
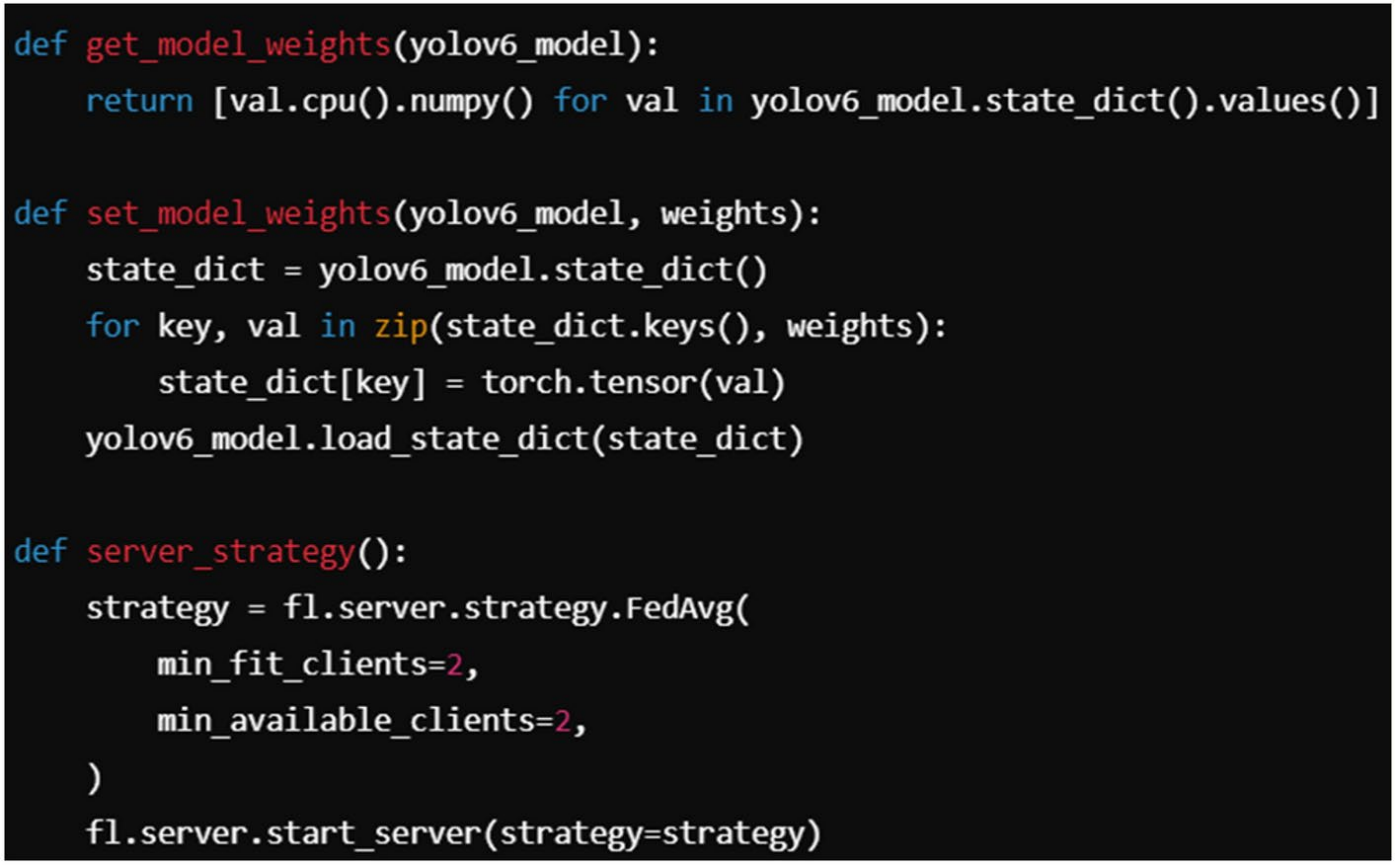
The snippet of code depicting aggregation of FedL with YOLOv6.

Integrating YOLOv6 with a Federated Learning (FedL) framework involves multiple steps that address the specific architecture of YOLOv6 and the decentralized nature of Federated Learning (which enables multiple clients to collaboratively train a model without sharing raw data). Yolov6 is a real-time object detection model that requires large amounts of labeled data for training. It uses convolutional layers for feature extraction and prediction. FedL is a decentralized learning framework that allows multiple devices (clients) to train models locally on their data and then aggregate the updates (model weights or gradients) on a central server without sharing the actual data. For integrating YOLOv6 and federated learning, the PySyft Pytorch library is used. In FedL, YOLOv6 will need to be trained decentralized across multiple clients. The clients will each maintain and update a copy of the YOLOv6 model using their local data. Each client trains the YOLOv6 model using local data without sharing the data with the server. After local training, the weights or gradients of YOLOv6 are sent to a central server that aggregates these updates using the FedAvrg method. The YOLOv6 model is initialized on each client. The pre-trained weights are loaded. Each client trains a copy of the YOLOv6 model on local data. After training, the local model’s parameters (weights) are sent to the central server. The server aggregates the weights received from multiple clients using an aggregation algorithm (i.e., FedAvrg). The aggregated model is then sent back to the clients for the next round of local training. Each client has its dataset of images used to train the YOLOv6 model locally. No raw image data is shared between clients. Only the model weights are communicated to the central server. FedL coordinated the training loop by sending a global model to each client. Clients train locally on their data and return the server’s model updates (weights/gradients). The server aggregates the updates and returns the updated global model to the clients for the next round. A snippet from the code is depicted in Fig. 3.

Some challenges must be addressed despite the advantages of integrating YOLOv6 with federated learning. YOLOv6 models are large, so exchanging model weights between clients and servers could become slow. This challenge is known as communication overhead. In FedL, clients may have non-IID (non-independent and identically distributed) data, making training more difficult. This challenge is known as data heterogeneity. While FedL provides more privacy than traditional methods, additional techniques like differential privacy or secure aggregation may be needed to enhance security. This challenge is known as Privacy. To overcome the challenge of communication overhead, model pruning is done. YOLOv6 has been pruned to remove unimportant weights or layers in the model. Model pruning is explained in a further section. To tackle data heterogeneity, some local data augmentations are made on clients, such as random rotations, FedLips, and scaling of images. While Federated Learning inherently improves privacy by not sharing raw data, it still leaves room for potential attacks (e.g., model inversion attacks) where attackers can infer sensitive information from model updates. To improve privacy, homomorphic encryption and decryption techniques are proposed that enable the server to aggregate model updates. Homomorphic encryption and decryption are explained in the previous sections.

For aggregation, the aggregator collects all the weight matrices from all the clients. The aggregator calculates the average weight value from the encrypted weights shared by all clients. This average weight value is considered the aggregate weight W_ag_ and is shared with all local clients for updating. Algorithm 5 shows the overall process for model aggregation.

**Algorithm 5:**
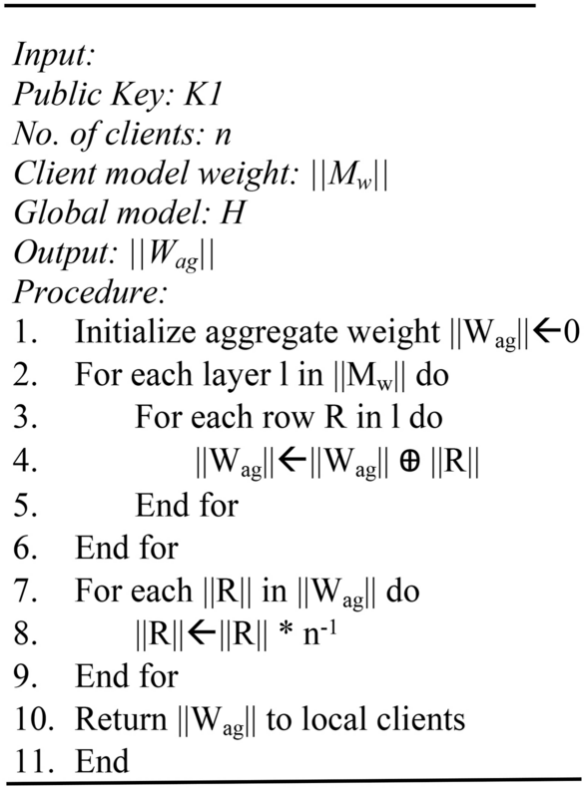
Model aggregation.

### Ensembled federated learning pruned YOLOv6

The architecture of YOLOv6 builds on the success of its predecessors in the YOLO family but incorporates various modern techniques for improving accuracy, efficiency, and speed, particularly for object detection tasks. YOLOv6 is designed to be highly optimized for real-time object detection, featuring a balance of model size and performance. The backbone of YOLOv6 is responsible for feature extraction from the input image. It transforms the raw pixel values into a feature map that contains high-level semantic information. The backbone of YOLOv6 uses CSPNet or similar variants to split the feature map into two parts, processing one part while keeping the other part intact. This helps to reduce computational complexity and memory usage while preserving spatial information. YOLOv6 incorporates standard convolutions, depthwise separable convolutions, and inverted residuals to extract features in a highly efficient manner, inspired by models like EfficientNet and MobileNet. The backbone includes residual blocks, which are inspired by ResNet, to enable deep feature extraction while preventing vanishing gradients during training. These modules adaptively recalibrate channel-wise feature responses, allowing the network to focus on important features.

The neck is responsible for aggregating features from different layers of the backbone and refining them for better object detection. YOLOv6 uses FPN to aggregate features from different scales. FPN combines high-resolution, low-level features with low-resolution, high-level features, allowing the model to detect both small and large objects effectively.

The head is responsible for predicting the bounding boxes, objectness scores, and class probabilities for the detected objects. YOLOv6 uses anchor boxes to predict bounding boxes at multiple scales. These anchors are predefined boxes of various sizes and aspect ratios that help in detecting objects of different sizes. The head predicts bounding box coordinates (x, y, width, and height) relative to the anchors for each object in the image. YOLOv6 predicts an objectness score for each anchor box, which indicates the likelihood of an object being present in that region.

This study uses a lightweight pruned YOLOv6 algorithm^32^ by the global model and the local clients to predict breast cancer in histopathological images. The pruned YOLOv6 helps to reduce the parameters and network depth^32^. Pruning is used to reduce the model’s weight and make it lightweight. Pruning helps remove those filters that are not required and hence improves the overall efficiency of the model. In^32^, the authors changed the optimizer of YOLOv6 from SGD to Adam and the learning rate from 0.01 to 0.0032.

This research employs a hidden layer pruning technique to make YOLOv6 a lightweight network by lowering the number of features and the network depth. In this type of pruning, entire layers or parts of layers are removed that have having least contribution to the performance of the model. In this process, the following steps are utilized.

Step 1: Begin the pruning process.

Step 2: Load the pre-trained model.

Step 3: A pruning criterion is defined. Normally it is defined as a minimum number of samples maximum depth, or minimum impurity decrease, but in this study, the hidden layers are defined as the pruning criteria.

Step 4: All the layers are iterated to check whether they meet the pruning condition or not with different values of M_i_, W_i_, and layer.

Step 5: If a layer meets the condition, it is pruned otherwise the layer is kept.

Step 6: Determine whether further pruning is possible or not. If possible, then continue evaluating layers and pruning them.

Step 7: The model is modified to reflect the changes and the performance of the pruned model is assessed to ensure it meets the desired metrics.

Step 8: The final pruned model is saved and this concludes the process.

**Fig. 4:**
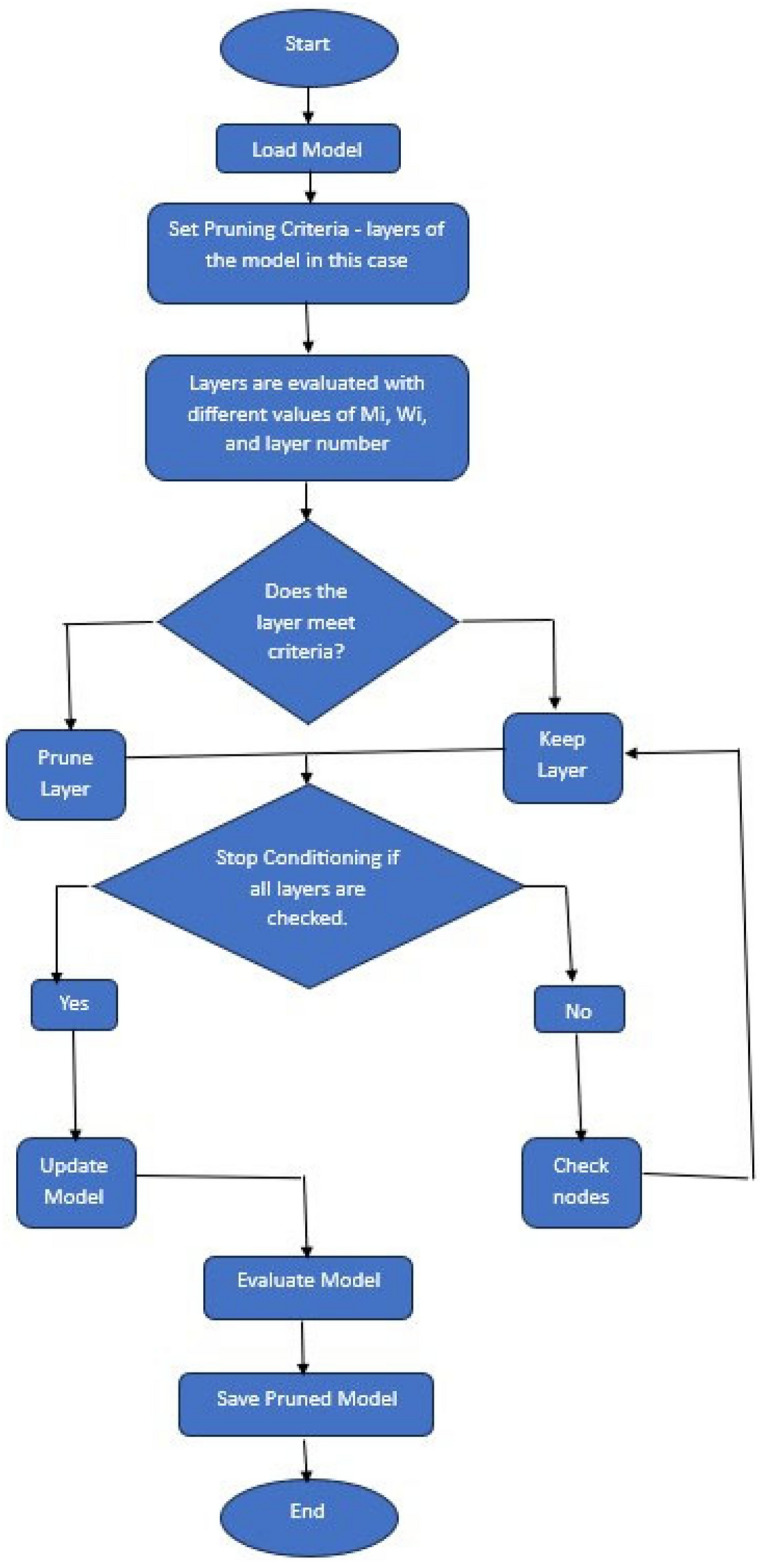
The flowchart for pruning process used to refine YOLOv6.

The flowchart to understand Algorithm 6 is depicted in Fig. 4.

Model trimming results in a drop in detection accuracy. A transfer learning technique was applied to overcome this situation to boost the detection accuracy. In this paper, the entire algorithm is thoroughly detailed. The algorithm is summarized as follows:

**Algorithm 6:**
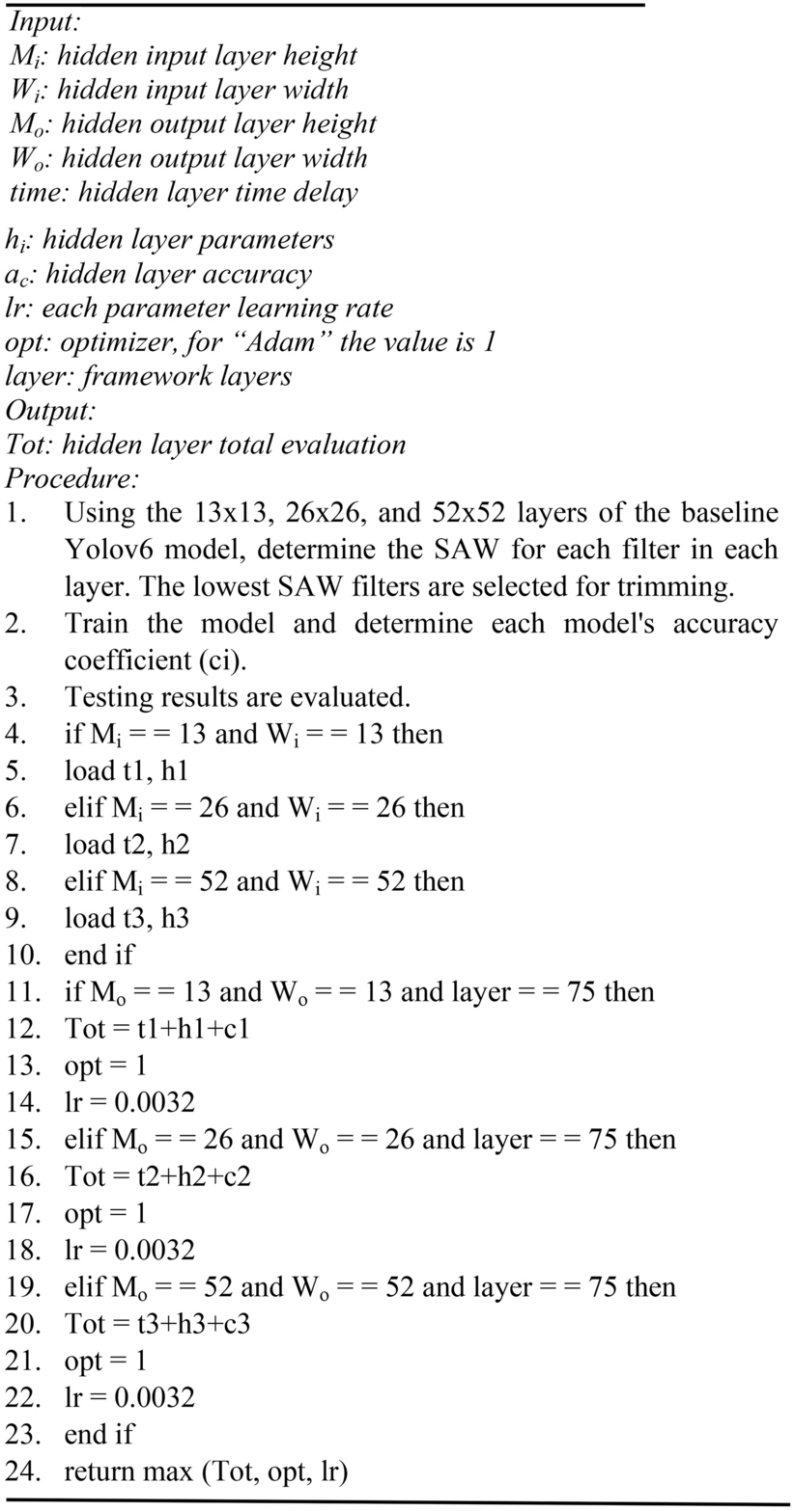
Pruning algorithm^32^

When integrating hidden layer pruning with federated learning (FedL), this optimization can help improve the system’s overall performance in several ways, particularly in reducing the computational and communication overhead. Pruning hidden layers in YOLOv6 reduces the number of model parameters. This results in a smaller model size, which directly impacts federated learning, where model updates (weights and gradients) are sent between clients and the central server during training rounds. In FL, clients periodically send their model updates to the server, which can lead to significant communication overhead. By pruning unnecessary neurons or filters from YOLOv6’s hidden layers, the size of these updates is reduced, minimizing the amount of data transferred between clients and the server. Pruned models require less data to be transmitted, which means faster communication between clients and the central server. Pruned YOLOv6 models are computationally less intensive, as fewer neurons or filters are involved in the forward and backward passes during training. In a federated learning setup, where each client trains the model locally before sharing updates, this means that client devices (those with limited computational power like edge devices or mobile healthcare units) can train the model more efficiently.

This study ensembles Pruned YOLOv6 with federated learning. This study uses the FedAvrg technique for aggregation. This study simulates federated learning across several devices but only occurs on a single computer. Algorithm 6 conducts local iterations. Therefore, after local training of the model, a global aggregate of these local models is created, disseminated for subsequent iterations, and so forth. This indicates that many communication rounds are required before a sizable portion of the local data is tagged. Thus, one option is to delay the communication rounds until the model has sampled a specific number of images. Because of this, only the first iteration is used to train the feature extraction layer. After training the feature extraction layer, the model’s core is trained similarly. The backbone of YOLOv6 remains frozen; hence, weights need to be updated, which reduces training time. The backbone data is not shared in communication rounds; therefore, it also saves bandwidth. Each model updates the backbone on its own as per the algorithm. Each client should be able to contribute as much as possible to the feature extraction to do this. One client trains the model with the labeled images and produces the weights. Then, another client reuses the same model and trains it with its local data. This keeps on repeating itself for a significant number of epochs. This enables clients to have a substantial impact on training. Hence, from all the models, the best ensembled federated weights are collected and shared with the global aggregator to create an aggregated model and update it appropriately. Algorithm 7 shows the overall procedure.

**Algorithm 7:**
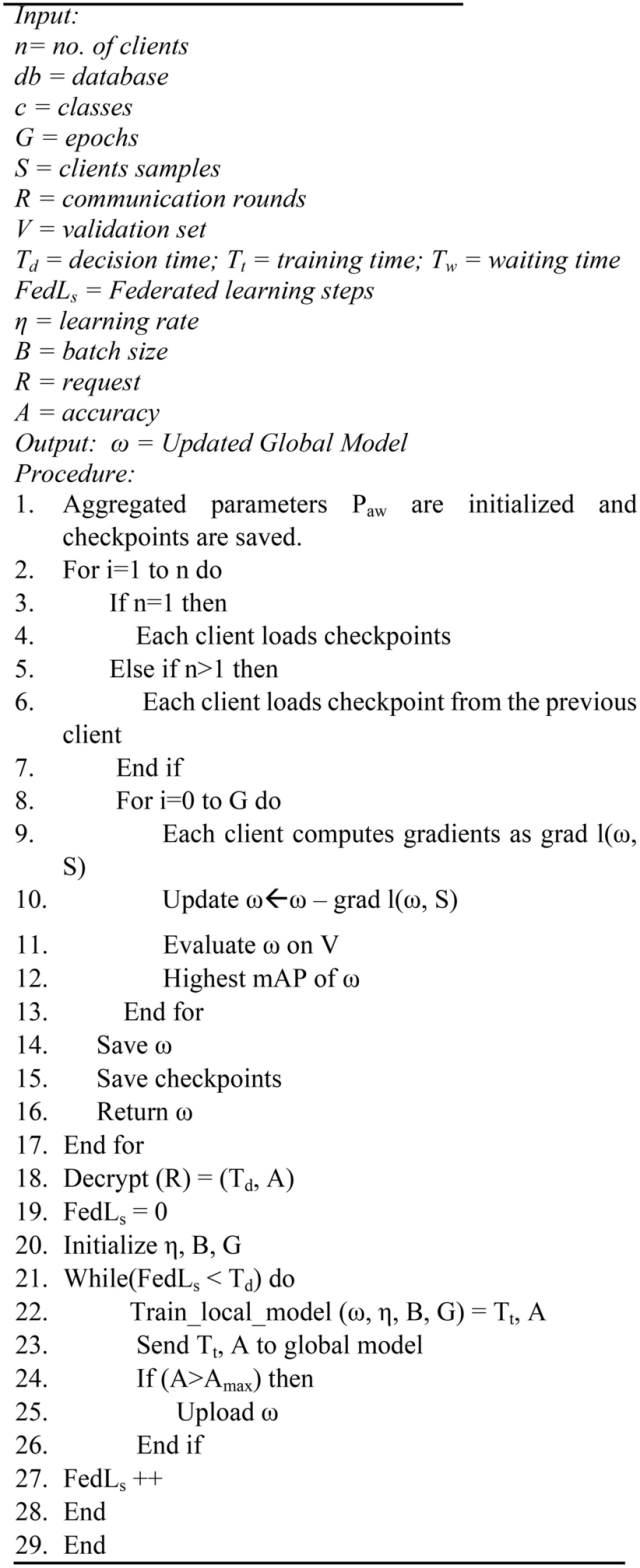
Ensembled FedL YOLOv6.

In YOLOv6, handling class imbalances in the dataset, mainly when certain classes are underrepresented, can be addressed through various techniques like data augmentation, class weighting, ensemble learning, and transfer learning. In this study, the pruned YOLOv6 is transferred and learned for handling class imbalances. Transfer learning is the process of Leveraging pre-trained models on larger datasets or related tasks that can help improve the performance of underrepresented classes. By fine-tuning it on the target dataset with imbalances, the model can profit from the knowledge transmitted from the pre-trained model and adapt to the unique features of the data. The transfer learning algorithm is modified, as Chhaya Gupta et al. stated in a research paper^32^. The same algorithm is used in this study with pruned YOLOv6.

The different parameters of the proposed model are categorized into different groups:

- YOLOv6 architecture and pruning parameters: These refer to the core object detection parameters in YOLOv6, and how pruning is applied to reduce the model size while maintaining performance. Different parameters involved are, input image size; backbone; head; anchor boxes; number of classes etc.
- Federated Learning parameters: These parameters define how the federated learning system operates, including communication and aggregation between clients (hospitals or medical institutions). These include number of clients; aggregation model; communication rounds; client selection; learning rate; batch size etc.

Table 1 provides the values of the hyperparameters utilized in this study.

**Table 1:** Parameters of proposed model.

Hyperparameters	Values
Image size	640 × 640 px
Batch size	32
Learning rate	0.01 and then 0.0032
Optimizer	Adam
No. of classes	2 (benign, malignant)
No. of clients	5
Aggregation model	FedAvrg
Client selection	Random
Epochs	100
Loss function for YOLOV6	Binary cross entropy

## Experimental results and discussions

The experiment was carried out in Google Colaboratory, where GPUs can be used on any machine with the help of this collaboration. The model is compared with the pre-trained models VGG-19, ResNet50, and InceptionV3 to classify breast cancer in histopathological images. Comparing the performance of YOLOv6 with VGG-19, ResNet-50, and InceptionV3 provides valuable insights into the strengths, weaknesses, and trade-offs of different deep learning architectures in the context of object detection tasks. It informs model selection, architecture design, and optimization strategies, ultimately contributing to object detection research and application advancement. Benchmarking the performance of YOLOv6 against well-established architectures like VGG-19, ResNet-50, and InceptionV3 serves as a reference point for assessing the state-of-the-art in object detection. The dataset has three sets: training, validation, and testing. Models are trained on the training set, and each model’s performance is calculated on the validation set. The local clients and global models have the same datasets and models with federated averaging techniques. All the models run for the same number of epochs. In this experiment, the models are trained for 100 epochs.

The model’s performance is trained and assessed using the BreakHis dataset in this experiment. The BreakHis dataset (Breast Cancer Histopathological Image Classification) is widely used in medical image analysis to classify breast cancer subtypes based on histopathological images. It provides high-resolution microscopic images of breast tissue samples, categorized into benign and malignant tumor classes. The images in the BreakHis dataset were obtained through microscopy of breast tissue biopsies, which are histopathological images. The dataset contains images showing tissue samples stained with Hematoxylin and Eosin (H&E), common stains used in pathology to highlight cell structures. The dataset is divided into two classes: benign and malignant. The collection includes 7909 pathological breast cancer images that were gathered from 82 people; 2480 are benign images, and 5429 are malignant images. The images are in high resolution, and each is 700 × 460 pixels. The large image size helps retain fine details, which are crucial for histopathological diagnosis. The dataset consists of images with magnifications like 40×, 100×, 200×, and 400×, as shown in Table 2. These different magnifications allow researchers to explore how well their models can handle various detail scales, which is essential for medical image analysis. The dataset was built by the RD laboratory in Brazil. This dataset consists of two classes, benign '0' and malignant '1'.

**Table 2:** Distribution of dataset.

Magnification	Malignant	Benign	Total
40×	1370	652	1995
100×	1437	644	2081
200×	1390	588	2013
400×	1232	588	1820
Total	5429	2480	7909

Distributing the BreakHis dataset among three clients in federated learning (FEDL) involves splitting the dataset while considering factors like class balance, data heterogeneity, and client capacity. Each client will train its model locally using its portion of the data, then send model updates (rather than raw data) to a central server for aggregation. Each client gets a subset of the data that reFedLects different distributions, simulating real-world scenarios where data might vary across clients (e.g., different hospitals or clinics). each client receives a non-uniform distribution of data. This is a more realistic scenario for federated learning, as clients often have data that reFedLects their specific environments, which leads to different distributions of classes, subclasses, or magnifications. Client 1 receives a majority of benign images and fewer malignant images.; Client 2 receives most of the malignant images; Client 3 receives a more balanced distribution of benign and malignant images, but only at low magnifications (40×, 100×); Client 4 receives a more balanced distribution of benign and malignant images, but only at high magnifications (200×, 400×); Client 5 receives a highly unbalanced subset focused on specific tumor subtypes. In this study, the non-IID (heterogenous) distribution technique is considered. The dataset is split uniformly among all five clients in a non-IID scenario. Non-IID mimics real-world situations where data distribution is uneven across clients. These strategies are critical for developing robust federated learning models, especially in medical applications like breast cancer diagnosis, where data heterogeneity reFedLects real clinical environments.

The model’s performance is trained and assessed using this experiment’s two datasets, BreakHis and BUSI. The BUSI dataset consists of 780 images with the same magnifications as BreakHis. The BUSI dataset (Breast Ultrasound Images Dataset) is a commonly used breast cancer diagnosis dataset focusing on ultrasound imaging. Breast ultrasound is a non-invasive imaging technique to detect abnormalities, such as tumors, in breast tissue. The images in the BUSI dataset are collected through ultrasound imaging, widely used in detecting breast cancer due to its effectiveness in distinguishing between different types of breast tissue. BUSI has a total of 780 images with 437 benign classes and 343 malignant classes^34^.

**Fig. 5:**
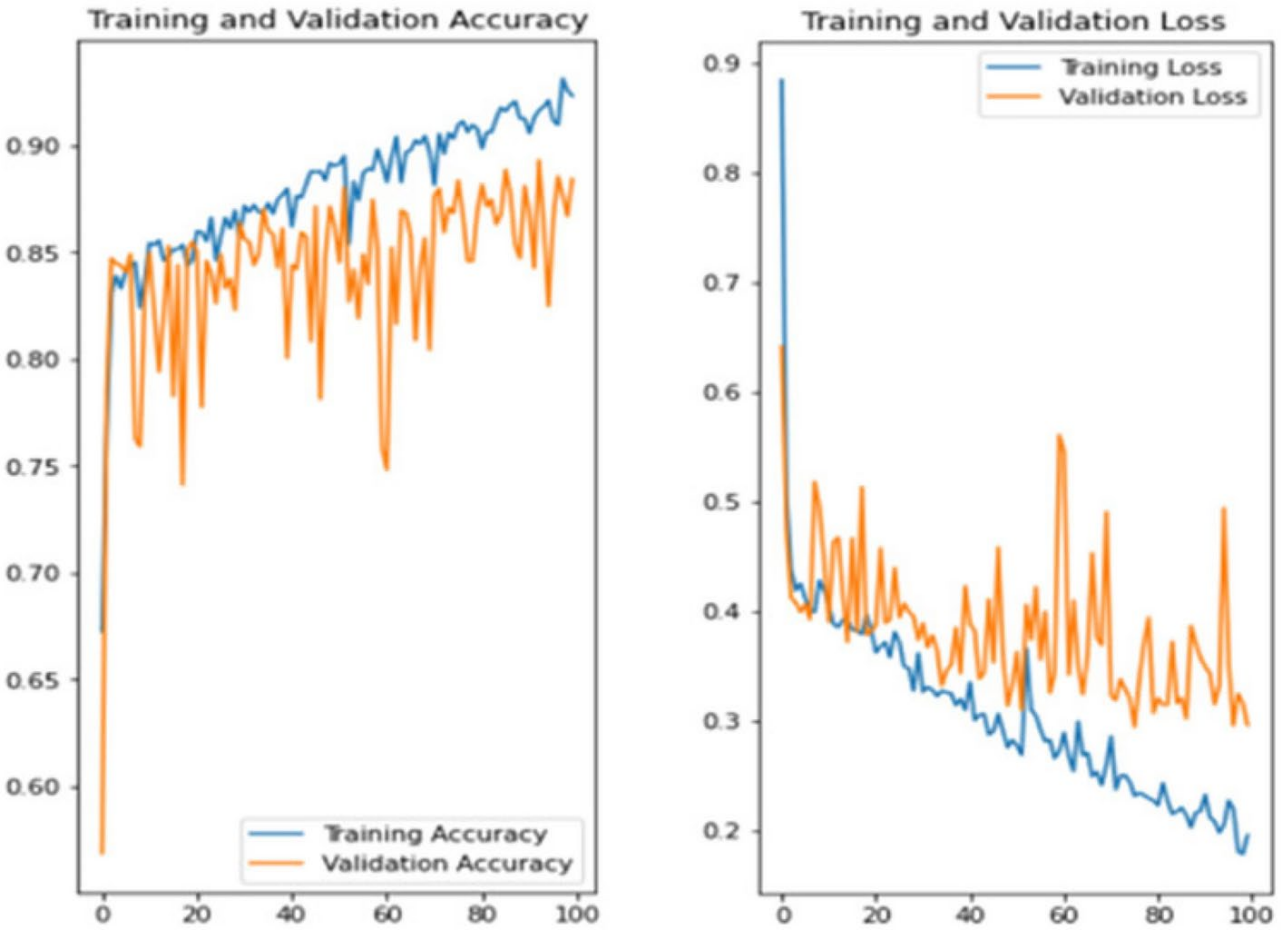
Accuracy and loss of ResNet50 model on BreakHis dataset after 100 epochs, depicting how accurately the model provides the results and how much loss it deals with.

**Fig. 6:**
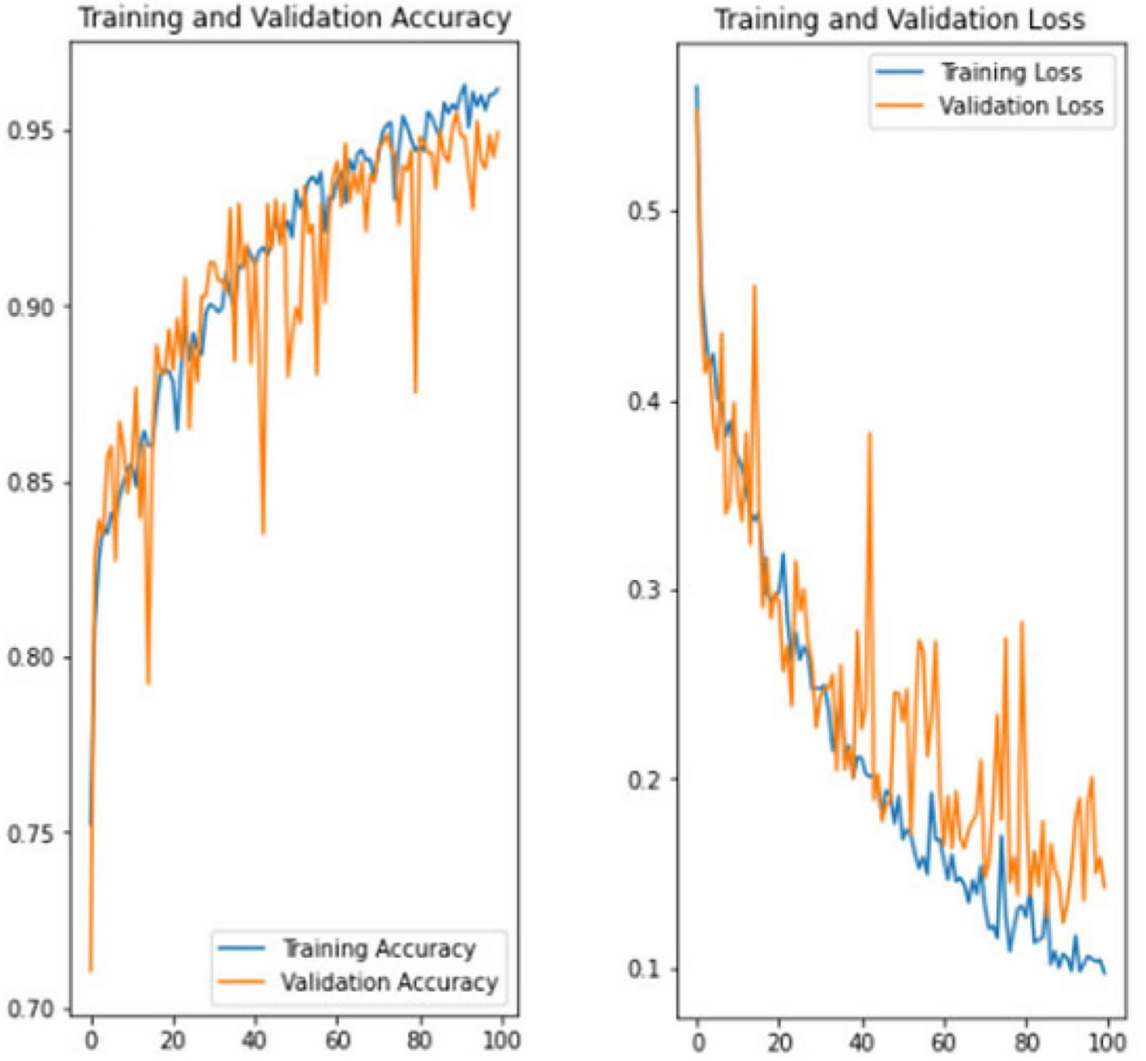
Accuracy and loss of ResNet50 on BUSI dataset after 100 epochs depicting how accurately the model provides the results and how much loss it deals with.

All the models are trained on these datasets. Pre-trained models like VGG-19, ResNet50, and InceptionV3 are available in the KERAS Python library. The dataset is partitioned with 80% as the training set, 10% as the validation set, and the rest as the testing set. In this experiment, three virtual local clients are used. In this experiment, non-IID partitioning is used. The evaluation metrics used are accuracy, loss, validation accuracy, and validation loss for each model. Table 3 depicts the accuracy (Ac), loss (L), validation loss (V_L_), and validation accuracy (V_A_) with 100 epochs with the ResNet50 model on the BreakHis dataset, and Table 4 depicts the results of the ResNet50 model on the BUSI dataset. Figure 5 depicts the accuracy and loss of ResNet50 on the BreakHis dataset. Figure 6 depicts the accuracy and loss of ResNet50 on the BUSI dataset.

**Table 3:** Performance results on BreakHis with ResNet50.

Epochs	A_c_	L	V_A_	V_L_
1	81.48	44.43	84.50	41.34
10	85.23	36.22	78.89	42.45
20	87.55	31.34	87.02	34.34
30	89.23	26.45	85.79	33.20
40	88.91	27.45	87.54	34.21
50	90.91	21.36	84.69	39.67
60	93.29	18.24	84.25	36.16
70	91.55	21.56	89.47	32.68
80	93.88	15.77	88.79	30.12
90	94.03	15.11	90.89	25.24
100	94.89	11.89	91.21	26.45

**Table 4:** Performance results on BUSI with ResNet50.

Epochs	A_c_	L	V_A_	V_L_
1	71.48	41.42	74.51	38.34
10	75.24	38.23	77.88	41.43
20	77.53	35.34	78.02	34.34
30	79.23	26.45	79.79	32.20
40	81.91	27.35	83.54	31.21
50	85.92	25.36	85.67	32.67
60	88.29	21.24	89.25	31.16
70	90.45	20.45	89.57	30.67
80	91.85	18.75	90.54	29.12
90	93.13	15.15	90.88	27.24
100	93.88	13.89	91.20	26.34

**Fig. 7:**
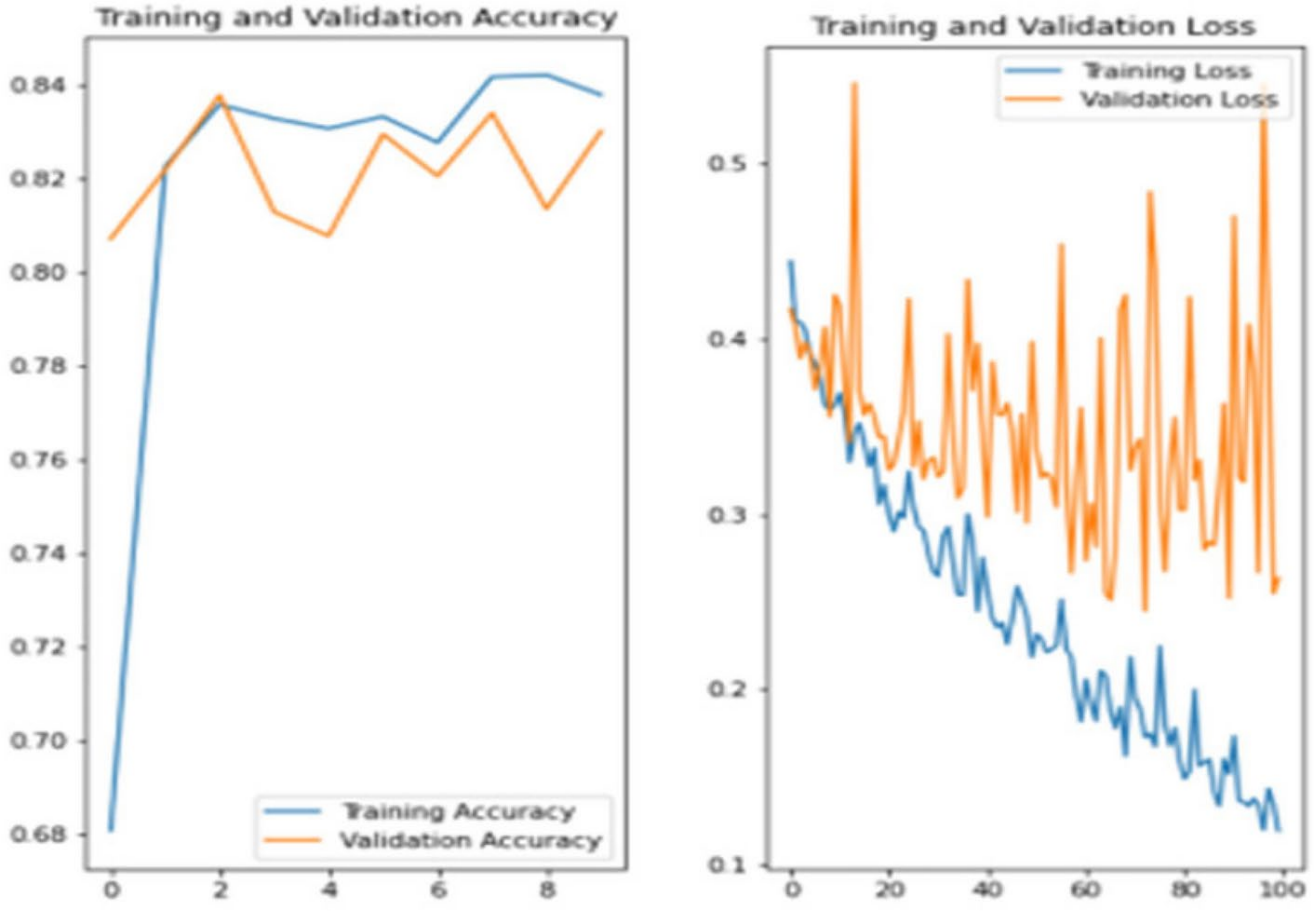
Accuracy and loss of VGG-19 on BreakHis dataset after 100 epochs, depicting how accurately the model provides the results and how much loss it deals with.

**Fig. 8:**
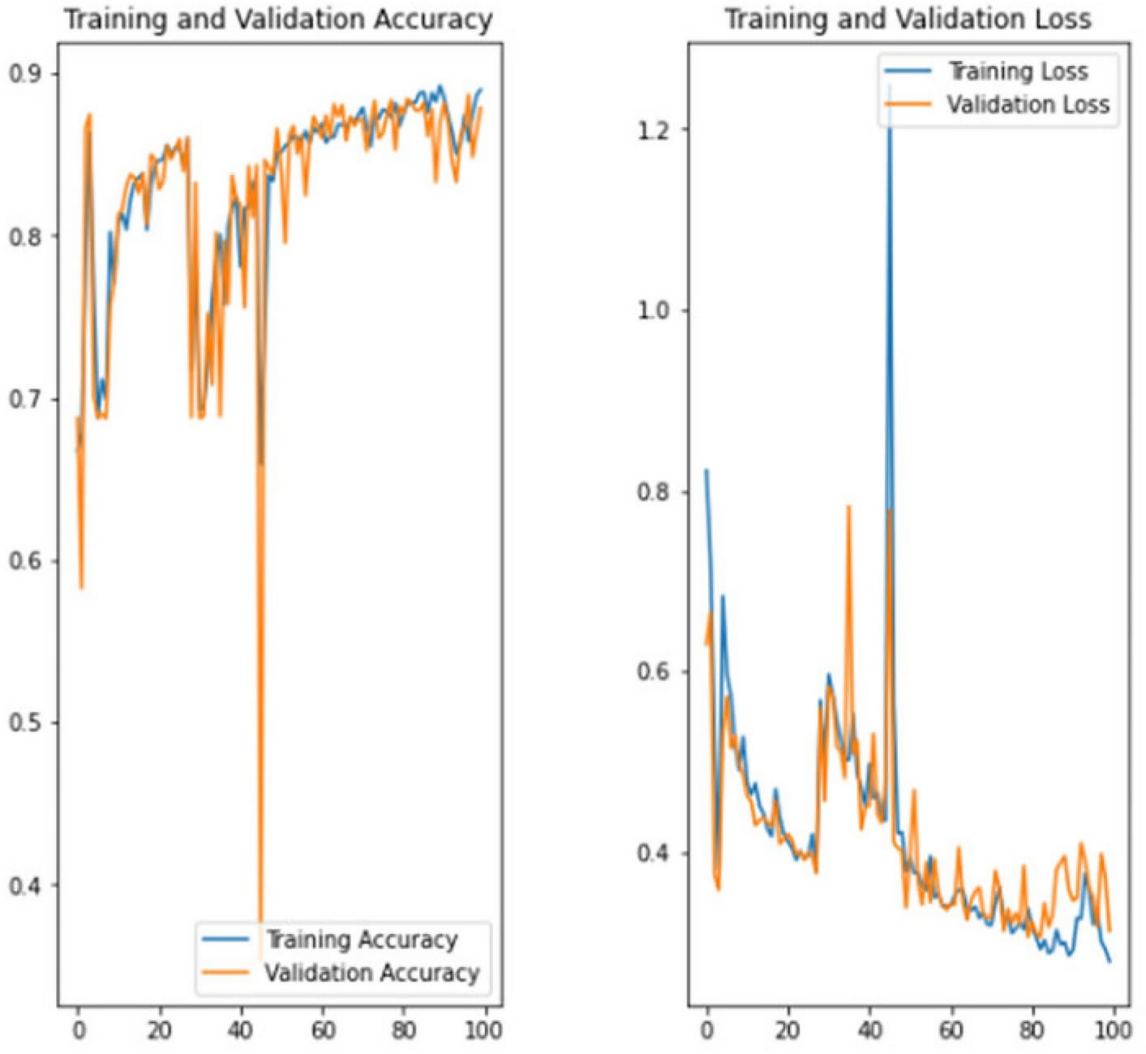
Accuracy and loss of VGG-19 on BUSI dataset after 100 epochs depicting how accurately the model provides the results and how much loss it deals with.

Tables 5 and 6 depicts the accuracy (Ac), loss (L), validation loss (V_L_), and validation accuracy (V_A_) with 100 epochs with the VGG-19 model on BreakHis and BUSI datasets. Figure 7 depicts the accuracy and loss of VGG-19 on the BreakHis dataset. Figure 8 illustrates the accuracy and loss of VGG-19 on the BUSI dataset.

**Table 5:** Performance results on BreakHis with VGG-19.

Epochs	A_c_	L	V_A_	V_L_
1	88.43	57.21	64.53	56.89
10	83.76	41.34	82.88	44.45
20	84.73	38.78	85.52	38.20
30	85.75	36.17	86.59	37.67
40	87.90	30.87	80.08	42.67
50	89.26	28.67	86.53	33.45
60	89.24	27.57	75.68	56.60
70	89.67	25.95	80.45	49.96
80	90.12	22.57	86.99	31.62
90	91.35	21.65	88.43	29.79
100	93.89	11.90	83.24	27.90

**Table 6:** Performance results on BUSI with VGG-19.

Epochs	A_c_	L	V_A_	V_L_
1	78.41	55.23	68.53	46.89
10	73.76	48.84	74.88	44.56
20	74.63	44.78	78.52	42.25
30	78.75	42.17	80.59	37.54
40	82.91	39.87	80.88	33.65
50	85.25	37.67	84.53	31.45
60	89.44	32.57	84.68	30.69
70	88.66	28.95	85.45	28.96
80	90.11	25.57	86.54	25.62
90	90.85	20.65	87.43	24.79
100	92.88	15.90	88.35	22.90

**Fig. 9:**
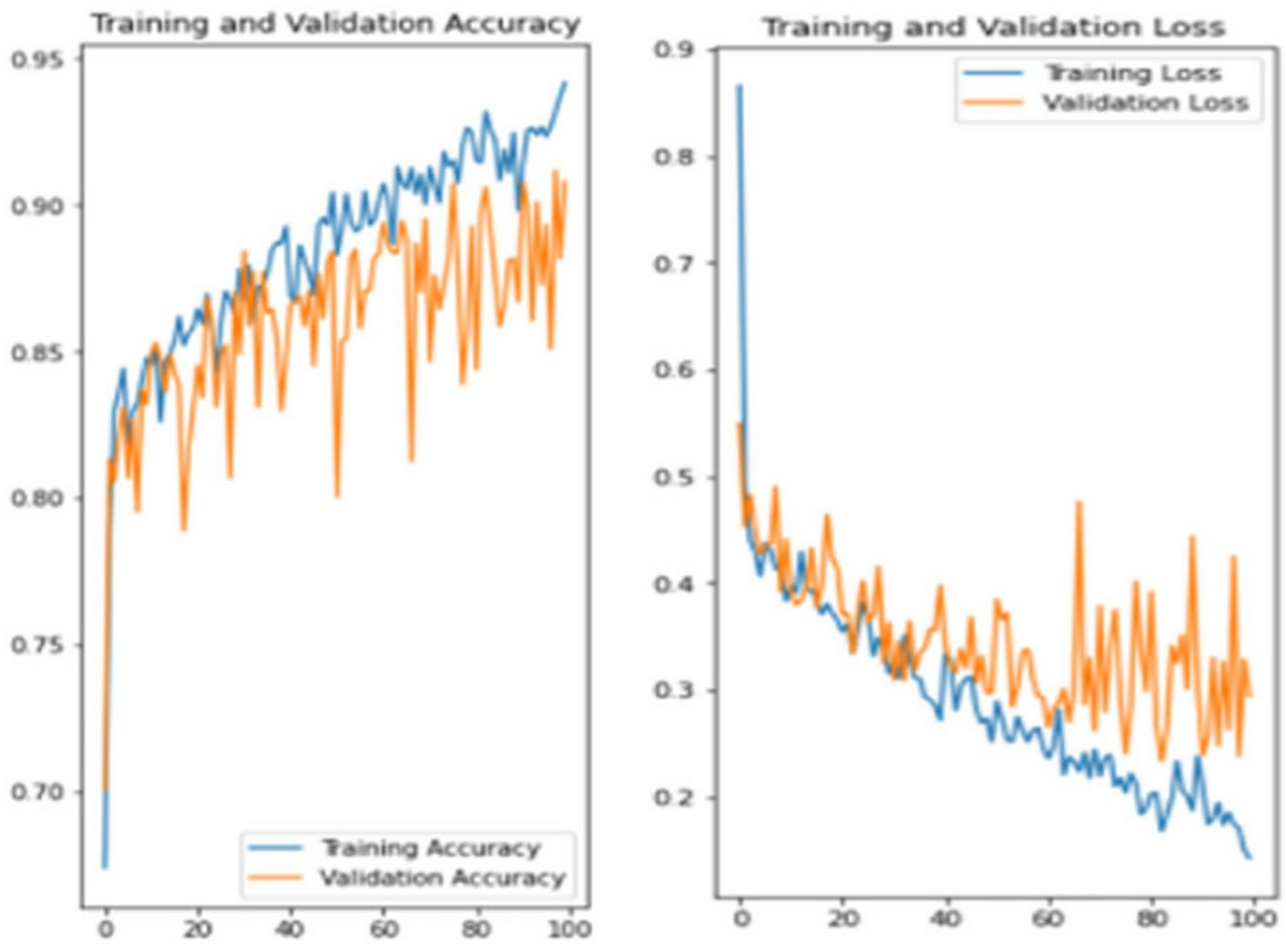
Accuracy and loss of Inception V3 on BreakHis dataset after 100 epochs, depicting how accurately the model provides the results and how much loss it deals with.

**Fig. 10:**
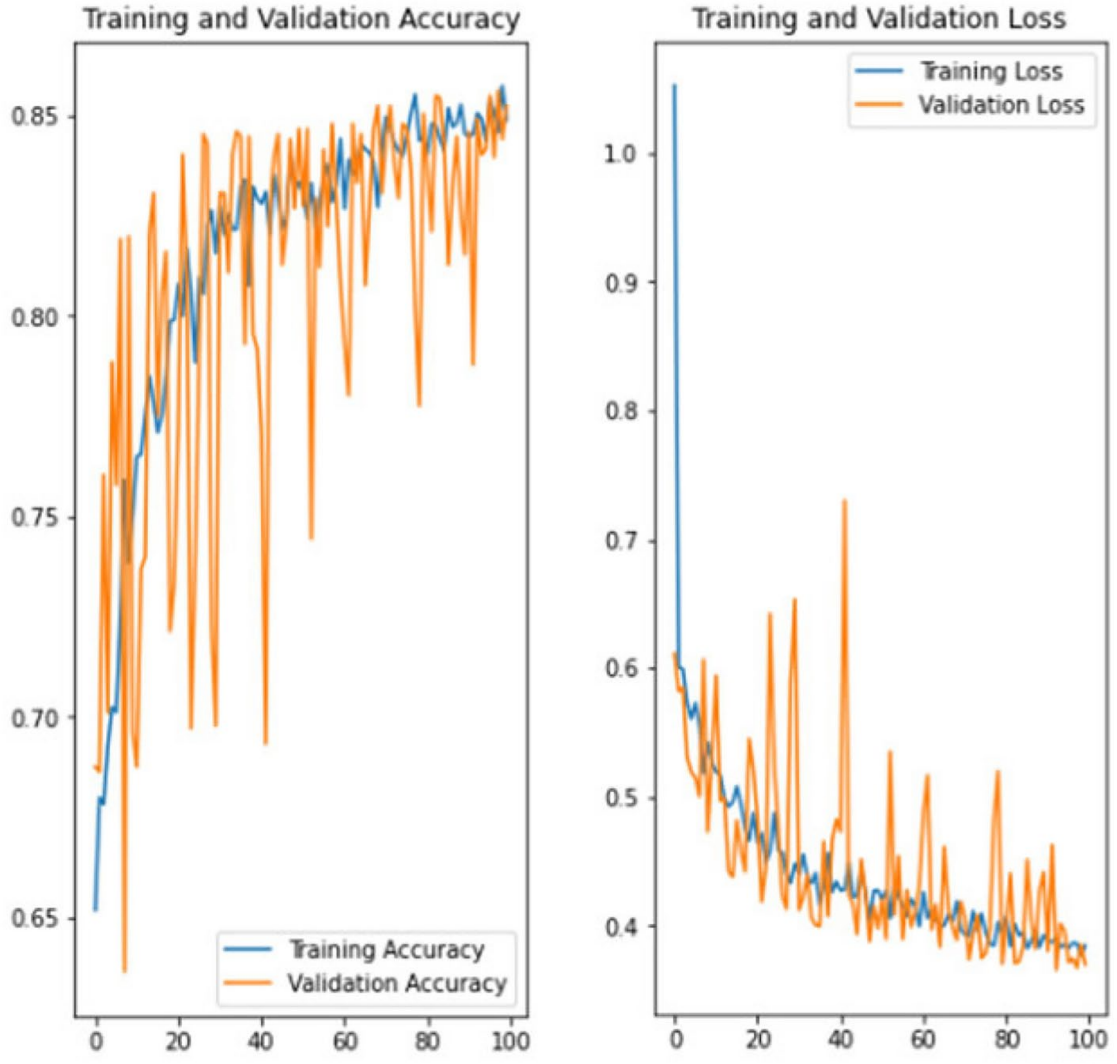
Accuracy and loss of Inception V3 on BUSI dataset after 100 epochs, depicting how accurately the model provides the results and how much loss it deals with.

Tables 7 and 8 depicts the accuracy (Ac), loss (L), validation loss (V_L_), and validation accuracy (V_A_) with 100 epochs with the Inception V3 model on BreakHis and BUSI datasets. Figures 9 and 10 depicts the accuracy and loss of InceptionV3 on the BreakHis and BUSI datasets.

**Table 7:** Performance results on BreakHis with Inception-V3.

Epochs	A_c_	L	V_A_	V_L_
1	67.32	56.54	70.12	54.89
10	84.35	38.38	83.19	55.23
20	85.56	36.57	83.08	41.53
30	87.89	31.37	84.92	36.74
40	89.64	27.18	84.88	39.54
50	89.31	25.91	88.34	29.67
60	90.71	24.52	88.73	28.52
70	90.05	24.67	89.25	26.91
80	92.64	18.89	89.72	29.68
90	89.39	23.66	86.71	30.71
100	94.71	14.52	90.85	20.93

**Table 8:** Performance results on BUSI with Inception-V3.

Epochs	A_c_	L	V_A_	V_L_
1	77.34	66.54	69.12	64.89
10	79.35	58.38	73.19	58.23
20	82.55	56.57	73.08	51.53
30	85.87	51.37	74.92	46.74
40	89.74	47.18	76.88	42.54
50	89.67	45.91	79.34	39.67
60	90.75	44.52	82.73	38.52
70	91.15	38.67	84.25	36.91
80	92.45	32.89	85.72	32.68
90	93.59	28.66	88.71	30.88
100	94.68	22.52	91.85	24.93

**Fig. 11:**
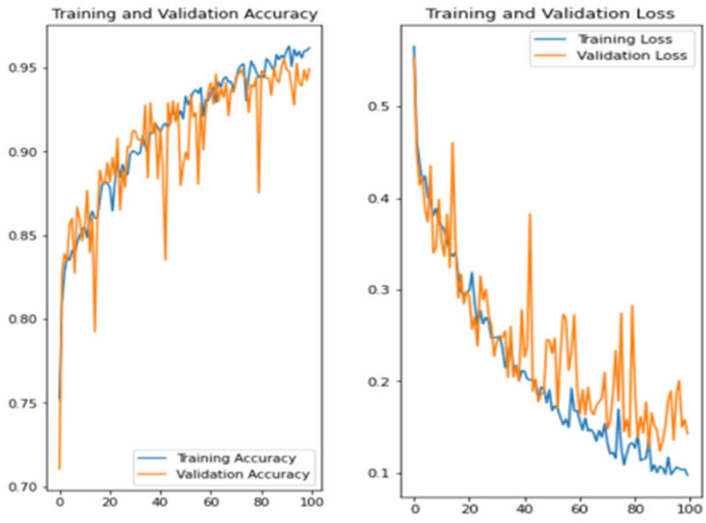
Accuracy and loss of proposed ensembled FedL YOLOv6 on BreakHis dataset after 100 epochs, depicting how accurately the model provides the results and how much loss it deals with.

**Fig. 12:**
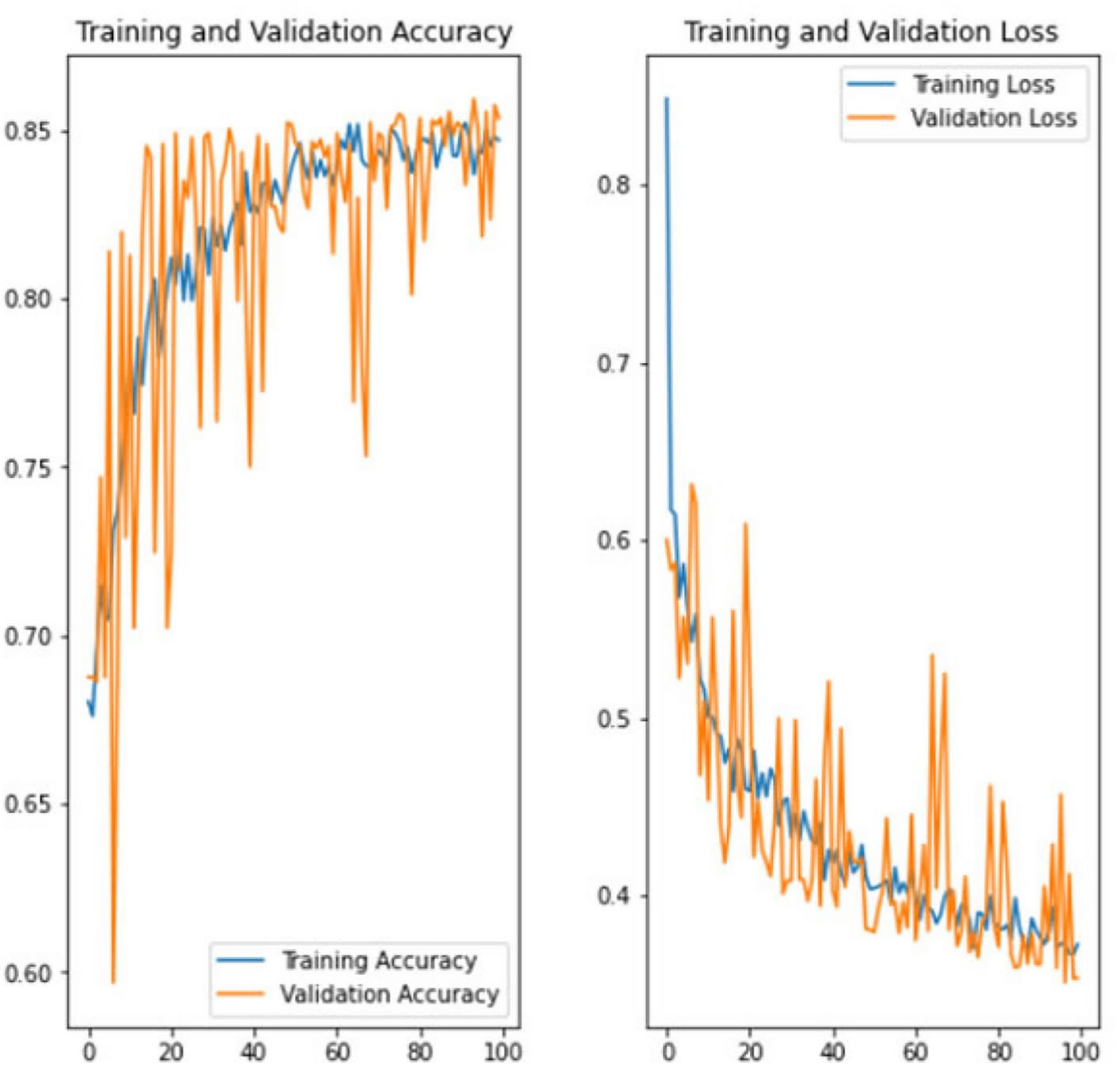
Accuracy and loss of proposed ensembled FedL YOLOv6 on BUSI dataset after 100 epochs, depicting how accurately the model provides the results and how much loss it deals with.

Tables 9 and 10 depicts the accuracy (Ac), loss (L), validation loss (V_L_), and validation accuracy (V_A_) with 100 epochs with the proposed Ensembled FedL Yolov6 model on BreakHis and BUSI datasets. Figures 11 and 12 depicts the accuracy and loss of the proposed model on the BreakHis and BUSI datasets.

**Table 9:** Performance results on BreakHis with proposed Ensembled FedL YOLOv6.

Epochs	A_c_	L	V_A_	V_L_
1	88.43	57.21	64.53	56.89
10	83.76	41.34	82.88	44.45
20	84.73	38.78	85.52	38.20
30	85.75	36.17	86.59	37.67
40	87.90	30.87	80.08	42.67
50	89.26	28.67	86.53	33.45
60	91.24	27.57	87.68	56.60
70	92.05	14.67	89.25	26.91
80	94.78	12.89	90.78	29.68
90	97.88	10.89	92.36	30.71
100	98.13	7.87	94.56	11.56

**Table 10:** Performance results on BUSI with proposed Ensembled FedL YOLOv6.

Epochs	A_c_	L	V_A_	V_L_
1	82.43	47.21	74.53	46.89
10	83.78	41.64	78.88	44.65
20	83.95	38.78	83.52	38.35
30	85.23	37.27	84.89	37.56
40	85.91	32.87	84.08	36.67
50	86.26	29.67	86.33	33.67
60	88.54	25.57	86.68	29.60
70	94.15	21.67	88.25	25.67
80	95.78	17.89	90.34	24.58
90	96.88	11.89	93.46	17.71
100	97.53	9.87	94.66	12.56

**Fig. 13:**
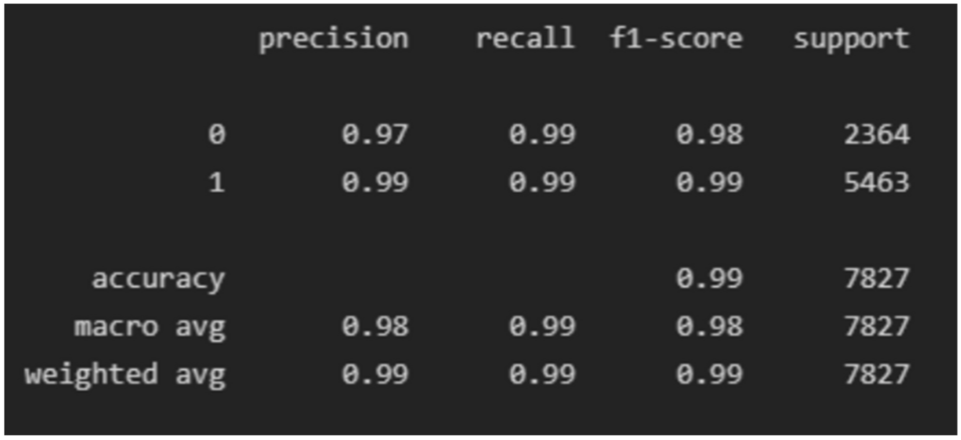
Classification report of the proposed model.

Figure 13 displays the proposed model’s classification report regarding precision, F1 score, and recall. This report aids in comprehending the suggested model’s overall performance. Specifically, True Positives, False Positives, True Negatives, and False Negatives are used to predict the metrics of a categorization report, as shown in the illustration below. Precision shows what percentage of predicted values were correct. Recall shows what percentage of positive cases were matched correctly. The F1 score depicts the percentage of positive predicted values that were correct. The model achieved a recall of 99% and an F1 score of 98%.

**Fig. 14:**
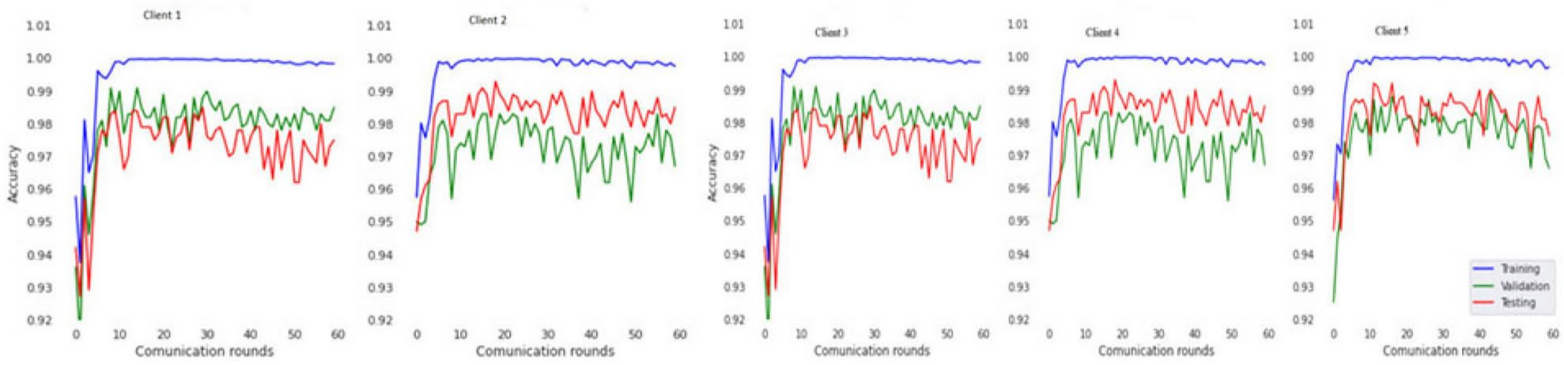
Accuracy of all five clients per communication round with the same settings.

The suggested model outperformed all other object detection models, with an accuracy of almost 98%, according to the data. Figure 14 depicts the accuracy of all five clients in different communication rounds with training, validation, and testing datasets. Table 11 provides a comparison with other SOTA models. The comparison is done based on the accuracy parameter. Only the accuracy metric has been considered as the SOTA models are considered for comparison and evaluated only on this metric.

**Table 11:** Comparison of proposed Ensembled FedL YOLOv6 with other sota models.

Models	Accuracy (%)
Lingxiao Li et al.^38^	91.06
Febrianti et al.^35^	84
Proposed	Approx. 98

The computational complexity of federated learning can vary depending on various factors, such as the size of the network, the number of participating clients (nodes), the complexity of the model, communication overhead, and the aggregation process. Here, some of the key factors influencing the computational complexity are outlined, out of which the computational complexity of federated learning is influenced by a combination of factors related to model complexity, communication overhead, aggregation processes, and security mechanisms:

- Model Complexity: The computational complexity of federated learning is directly influenced by the trained model’s complexity. More complex models with a more significant number of parameters typically require more computational resources for training.
- Number of Clients: The computational complexity increases with the number of participating clients in federated learning. Each client performs local computations on its data, and these computations need to be coordinated and aggregated at a central server or among clients, which adds to the overall computational load.
- Communication Overhead: Communication between the clients and the central server or among clients is a significant factor in federated learning. Transmitting model updates, gradients, or aggregated parameters incurs communication overhead, contributing to the overall computational complexity, especially in scenarios with limited bandwidth or high-latency communication channels.
- Aggregation Process: Aggregating model updates or gradients from multiple clients involves additional computational overhead, primarily when sophisticated aggregation techniques such as federated averaging, secure aggregation, or differential privacy mechanisms are employed. The computational complexity of the aggregation process depends on the number of clients, the size of the updates, and the chosen aggregation method.
- Local Computations: Each client performs local computations during training, including forward and backward passes through the model, gradient computations, and potentially other operations such as data preprocessing or augmentation. The computational complexity of these local computations depends on factors such as the size of the local dataset and the complexity of the model.
- Security and Privacy Mechanisms: If federated learning incorporates security and privacy mechanisms such as encryption, differential privacy, or secure aggregation, the additional computational overhead is incurred to perform these operations, potentially increasing the overall complexity of the system.

Implementing homomorphic encryption with federated learning (FedL) in real-world healthcare settings presents several practical challenges that must be addressed to ensure data security, scalability, and performance. These challenges arise due to the unique requirements of healthcare environments, such as strict data privacy regulations, resource constraints in clinical settings, and the need for real-time or near-real-time decision-making.

Deployment Challenges: Healthcare settings use various medical devices, hospital information systems (HIS), electronic health record (EHR) systems, and diagnostic tools. These systems may have different technical standards, hardware capabilities, and software architectures. Integrating FedL with homomorphic encryption into this diverse landscape is challenging. Many healthcare applications use edge devices like medical imaging machines (e.g., MRI, X-rays), wearables, or IoT devices for real-time data collection. These devices often have limited computational power, memory, and storage, making running resource-intensive processes like homomorphic encryption and local model training required in federated learning challenging. Federated learning involves multiple clients (e.g., hospitals, clinics, and medical research institutions) that collaboratively train a model while keeping data localized. As the number of participants grows, the system must be able to scale. However, federated learning with homomorphic encryption faces computational overheads that can bottleneck communication and aggregation processes, making it difficult to scale to large healthcare networks. Many healthcare institutions rely on outdated or legacy systems not designed to handle advanced machine-learning techniques or encryption protocols. Retrofitting these systems to support federated learning and homomorphic encryption would require significant investments in hardware, software upgrades, and infrastructure improvements.

Data Security: Homomorphic encryption allows computations to be performed on encrypted data, preserving privacy, but it comes with high computational overhead, as discussed earlier. Handling encrypted data introduces complexities in the storage and transmission processes. Healthcare organizations must ensure encrypted data is appropriately managed, stored securely, and transmitted efficiently between distributed nodes.

Computational Cost: In federated learning, each healthcare organization (or edge device) trains the model on its local data and encrypts updates before sending them to the central server. However, many clinical institutions or edge devices (e.g., in remote clinics) cannot access high-performance computational resources. This creates a bottleneck for training encrypted models or even applying encryption schemes at the local level. This increases the computational cost of the system. Real-time decision-making is essential in many healthcare applications, especially in critical care settings. For example, automated systems for detecting abnormalities in medical images (e.g., X-rays, MRIs) or predicting patient outcomes in intensive care units (ICUs) must provide timely results. The computational delays introduced by homomorphic encryption can make meeting these real-time or near-real-time requirements challenging.

To address the above-stated issues in real-world healthcare settings, it is essential to focus on strategies that balance data security, computational efficiency, and scalability while adhering to the requirements of healthcare environments. Healthcare devices (e.g., MRI machines and wearables) can act as edge devices for local data processing. The burden of centralized computation is reduced by performing local training on these devices and sending only encrypted updates to a central server. Deploy a middleware layer that interfaces federated learning and the various health information systems (HIS). This ensures that encrypted updates, model parameters, and data formats can be easily exchanged between systems, regardless of their architecture. Deploy FedL and homomorphic encryption in a limited, controlled clinical environment first (e.g., a single hospital or a small group of clinics) to identify specific integration issues. This allows institutions to test and refine the system without significant disruptions to day-to-day operations. Batched encryption can process multiple data points or operations simultaneously under a single encryption operation. Batching multiple operations reduces the computational cost per data point, making the encryption and decryption processes more efficient without sacrificing privacy. This study has applied this technique to reduce the number of encryptions and operations required. Using model compression techniques like pruning helps reduce the size of the transmitted encrypted model updates. This can reduce the overall computational burden on the client side and decrease communication costs between healthcare nodes and the central server. Pruning is also employed in this study to reduce the computational cost of the model.

**Fig. 15:**
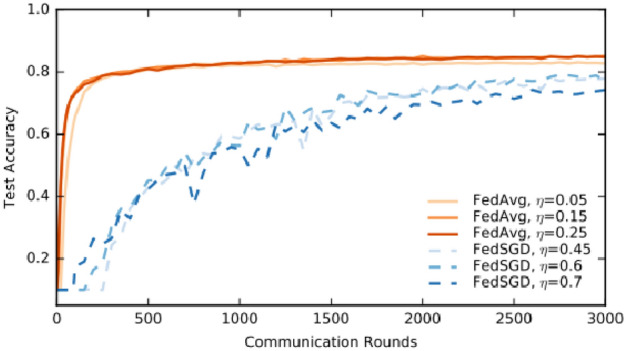
Learning rate curves for the proposed model.

Figure 15 shows the learning rate curves of the proposed ensembled FedL YOLOv6 model. The learning rates are depicted by η. From algorithm 7, it is clear that the suggested model is trained using different learning rates. The model indicates approximately equal accuracies with 0.25, 0.15, and 0.05 learning rates.

## Conclusion

The federated learning-based pruned YOLOv6 model with five clients, applied to histopathological images for brea**st** cancer diagnosis, presents a novel and practical medical image analysis approach while addressing critical privacy, scalability, and computational efficiency challenges. The model ensures privacy-preserving training by allowing each client (representing hospitals or medical institutions) to retain local control over sensitive breast cancer histopathological images. Training the YOLOv6 model in a federated setup combines knowledge from multiple clients, each with its own distinct dataset. Pruning the YOLOv6 model reduces its size and computational complexity, making it more feasible for local client training while maintaining object detection accuracy. Leveraging the FedAvrg algorithm to aggregate local updates, the model benefits from the diversity of data across different clients, leading to enhanced performance in detecting cancerous regions in histopathological images.

The tests reveal that federated learning is feasible, as FedAvrg trains models of outstanding quality with only a few communication rounds, as shown by the results on a range of model topologies such as ResNet50, VGG-19, InceptionV3, and the proposed Ensembled FedL YOLOv6. The model is also compared with some of the SOTA models. This study suggests a novel ensembled federated learning system that may perform knowledge fusion by aggregating model parameters while adhering to data privacy requirements. The aggregator in the system contributes to load management for multi-user access. The distributed computing framework affects the advantages of computing efficiency. The encryption techniques protect the confidentiality of requests and outcomes as the brain of the federated training system. The proposed model is used to classify breast cancer histopathological images. Four models have been compared in this study using a federated learning framework, showing impressive results. The proposed model offers the best results and achieves an accuracy of 98%. The proposed model provides the best results and achieves an accuracy of 98% when trained on the BreakHis dataset and an accuracy of 97% when trained on the BUSI dataset.

Utilizing an Ensemble Federated Learning YOLOv6 framework for breast cancer detection addresses data privacy, data variability, model robustness, and resource efficiency challenges while promoting collaboration and trust among healthcare institutions. Combining the strengths of federated learning, ensemble learning, and YOLOv6 architecture, this framework offers a promising approach to improving breast cancer detection accuracy and clinical outcomes.

The model has faced challenges due to non-iid (non-identically distributed) data across different clients, which could cause bias if not correctly handled. Communication cost and computational load are the other challenges the proposed model faces. Pruning and homomorphic encryption techniques are proposed in this study to handle these challenges.

In the future, the operating efficiency of the proposed homomorphic encryption algorithms will be improved so that data imbalance problems can be further resolved. Data imbalance is the main issue in terms of medical imaging. Other techniques will be implemented to resolve class imbalance for underrepresented datasets. With more extensive and well-balanced datasets from differently distributed and non-identical data sources, the trained network can be tested in subsequent research. Nonetheless, as data imbalance is a common issue in the medical industry, future research should be done on solutions. In addition, the practical performance of the complete federated learning framework—including its vulnerability to security breaches—and the efficiency with which the homomorphic encryption algorithms operate will also be measured.

## Data Availability

Data is freely available online at https://github.com/chhayagupta13/FEDL_BC.

## Declarations

### Use of AI tools declaration

The authors declare they have not used Artificial Intelligence (AI) tools in the creation of this article.
